# Systematically analyzing behavior change techniques used in 44 interventions to reduce unprofessional behavior between healthcare staff

**DOI:** 10.1093/tbm/ibaf058

**Published:** 2025-10-15

**Authors:** Justin Aunger, Bianca Ungureanu, Jill Maben, Ruth Abrams, Alice M Turner, Johanna I Westbrook

**Affiliations:** NIHR Midlands Patient Safety Research Collaboration, University of Birmingham, Birmingham, United Kingdom; Department of Applied Health Sciences, School of Health Sciences, University of Birmingham, Birmingham, United Kingdom; NIHR Midlands Patient Safety Research Collaboration, University of Birmingham, Birmingham, United Kingdom; Department of Applied Health Sciences, School of Health Sciences, University of Birmingham, Birmingham, United Kingdom; School of Health Sciences, Faculty of Health and Medical Sciences, University of Surrey, Guildford, United Kingdom; School of Health Sciences, Faculty of Health and Medical Sciences, University of Surrey, Guildford, United Kingdom; NIHR Midlands Patient Safety Research Collaboration, University of Birmingham, Birmingham, United Kingdom; Department of Applied Health Sciences, School of Health Sciences, University of Birmingham, Birmingham, United Kingdom; Australian Institute of Health Innovation, Macquarie University, Sydney, New South Wales, Australia

**Keywords:** behavior change techniques, health services research, behavioral science, professionalism, unprofessional behavior, patient safety

## Abstract

**Background:**

Behavioral and implementation science frameworks should be employed in the design of interventions to change behavior, including those delivered in organizational settings, to enhance their effectiveness, replicability, and transparency. However, this is often not done well in health services research. This deficiency also impacts interventions to address unprofessional behaviors (UBs) among healthcare staff. UBs include rudeness and bullying, which harm patient safety and staff wellbeing. This study builds on an earlier realist review of these UB interventions to retroactively identify their active components.

**Methods:**

A systematic search was updated to July 2024 using MEDLINE, Embase, CINAHL, and Google Scholar. Intervention descriptions were extracted from study reports and independently coded using directed content analysis against the May 2024 version of the behavior change technique (BCT) Ontology, which contained 284 BCTs.

**Results:**

The search identified 262 titles and abstracts, yielding five new reports. Combined with 42 papers from the prior review, 47 reports of 44 interventions were included. Interventions were categorized as single-session (*n* = 15), multisession (*n* = 12), combined session (*n* = 6), professional accountability (*n* = 7), and structured culture change (*n* = 4). Complex interventions used more BCTs: session-based interventions focused on awareness-raising and roleplay, professional accountability on consequences, and structured culture change on goal-oriented techniques. Few interventions reported negative outcomes, limiting the understanding of which BCTs drive effectiveness.

**Conclusions:**

The BCT ontology is broadly applicable to organizational behavior change in healthcare. Complex interventions employ consequence-based and goal-oriented BCTs, but the effectiveness of specific BCTs remains unclear due to poor evaluations. Future interventions should use the BCT Ontology to improve intervention reporting and effectiveness.

## Teaser

- This study reviewed ways to reduce unprofessional behaviors like rudeness and bullying among healthcare staff and identified key components of interventions seeking to improve workplace culture and patient safety.
- The research highlights the importance of using clear frameworks to design and report workplace behavior interventions, which will make it easier to understand what works while also seeking to create better healthcare environments for staff and patients.

Implications

- **Practice:** The behavior change technique ontology is broadly applicable to organizational interventions and should be used, even in organizational behavior change interventions, to improve understanding of how and why interventions may work, improve replicability, and improve the ability to synthesize these interventions.
- **Policy:** Policymakers should require publicly funded research to properly report interventions to improve replicability and improve research waste.
- **Research:** Interventions to reduce unprofessional staff behavior are still at an early stage of development; more robustly designed, evaluated, and reported interventions are needed to gain understanding of what intervention components are most effective.

## Introduction

Organizational interventions to modify staff behavior are reliant on successful behavior change; however, these types of interventions are not always conceptualized as “behavior change interventions” that necessitate use of behavioral science [1–3]. This means that interventions to improve aspects of healthcare organization often focus on altering processes, workflows, or organizational structures without explicitly recognizing these changes as forms of behavior modification. The fields of behavioral and implementation science present a wealth of frameworks and theories that can be drawn upon to aid behavior change, support effectiveness of an intervention, and improve reporting and replication of interventions in the healthcare intervention space [4]. Although these frameworks are widely used and essentially required in health-related behavioral research, unfortunately, use of behavioral science in organizational interventions in healthcare is still poor [4, 5].

Design of behavior change interventions *should* include several considerations such as the population targeted, mode of delivery (e.g. intervention format), theoretical foundations (e.g. COM-B, theory of planned behavior), and use of behavior change techniques (BCTs), among others [1]. Logic models should also be used while drawing on these behavioral science concepts, to set out how interventions are intended to work, why, what assumptions need to be in place, and how the chosen outcomes assess these changes [6, 7]. BCTs are one important piece of this puzzle. BCTs can be defined as “coordinated sets of activities designed to change specified behavior patterns” [8]. An example of a BCT is the “goal-directed BCT” which can be defined [in the BCT ontology (BCTO)] as “a BCT that sets or changes goals” [9]. For example, a goal could be (in the case of a physical activity intervention), to walk 10 000 steps per day, every day. Several taxonomies of BCTs exist [8–10]. For example, the BCT Taxonomy v1 offers a standardized, hierarchically structured classification of 93 discrete techniques used to change behavior [8]. These techniques are organized into groupings based on their function (e.g. goals and planning, feedback and monitoring, social support), and are designed to be observable, replicable, and theory-linked. The BCT taxonomy aligns closely with the COM-B model for behavior change [11], which posits that behavior is influenced by capability, opportunity, and motivation. Each BCT can be mapped to one or more components of COM-B, providing a systematic way to select and report intervention content that targets specific behavioral determinants. More recently (as of 2024), the BCT taxonomy is being expanded to become the BCTO—which comprises 284 (and counting) BCTs [9].

BCTs can be delivered through different modes (e.g. verbally, via print, etc.), and work through different mechanisms of action (MoA). MoAs are defined as “the processes through which a BCT affects behavior” [12]. Understanding of BCTs has historically varied between intervention authors, as BCTs have not been operationalized or standardized until recently [13]. This undermined reporting of interventions leading to poor replicability in new contexts, poor ability to synthesize interventions, and difficulty in understanding what may make them effective [8]. Thus, use of a BCTO to describe an intervention is useful, because it can enhance standardization and clarity, facilitate understanding of “what was actually done” for those seeking to replicate such interventions, enable understanding of underlying MoAs for these BCTs, and can enhance synthesis in systematic reviews [8, 14].

One specific area within health services research in which there has been underuse of behavioral science, is in interventions seeking to address unprofessional behaviors (UBs) between healthcare staff [2]. We have previously defined unprofessional behavior as “any interpersonal behavior by staff that causes distress or harm to other staff in the healthcare workplace” [15]. Here, we mean for “healthcare staff” to include all clinical and nonclinical staff working in acute healthcare settings [15]. This term “unprofessional behavior” can be used to encompass behaviors such as rudeness, incivility, microaggressions, bullying and violent, and/or criminal behaviors such as sexual or physical assault. In 2023, we published a realist review of interventions to reduce UB in acute healthcare settings globally [2]. We identified 42 interventions which had been tested or evaluated in some form. However, we found that these interventions did not draw on any form of behavioral science, or even logic modelling, in their design. There was also poor use of theoretical frameworks of any kind, with only *n* = 18 (43%) reporting using any at all [2]. This is not uncommon—others have reviewed nonpharmacological interventions more widely to examine replicability and found that only 39% interventions were adequately described [16].

UB between healthcare staff negatively impacts staff wellbeing, patient safety, and generates substantial costs for organizations [17]. UBs appear to be endemic in the UK’s National Health Service (NHS) and globally [17]. Extreme occurrences of UB as well as prolonged “milder” UB such as incivility can both have significant impacts on patient safety, staff wellbeing, and organizational effectiveness [17]. Recent examples highlight the pervasiveness of UB. In April 2023, Professor Bewick’s investigation into the clinical safety in a UK trust “heard repeated reports of a longstanding ‘bullying and toxic’ environment” with negative impacts on patient care [18]. A large study of hospital staff in Australia (*n* = 5178) showed that staff recognized the impact of these behaviors on clinical team performance and quality [17]. Staff who experienced frequent incivility from colleagues were significantly more likely to report these behaviors negatively impacted quality of care [odds ratio (OR): 7.09, 95% confidence interval (CI): 5.17–9.73] and teamwork (OR: 10.02, 7.25–13.8) compared to those who had not experienced these behaviors [17]. An analysis of reports (*n* = 1310) made by staff about the UB of colleagues in eight Australian hospitals (*n* = 1310) found that the types of UB identified by staff as most commonly jeopardizing patient safety were opinions being ignored, withholding of information, and behaviors which negatively impacted handovers [19]. Given the association of UB with patient safety and staff wellbeing, reducing UB could provide significant benefits to health systems. Understanding precisely and objectively what intervention components are, and how effective they may be, is essential from the design stage of these interventions, through to reporting of results.

Performing a retrospective analysis of the implicit BCTs used in existing interventions to reduce UB can: (i) improve understanding of existing interventions, (ii) demonstrate that drawing on behavioral science frameworks is possible for these interventions, (iii) encourage uptake of these frameworks, and (iv) may assist in identifying and understanding how future interventions (and which BCTs) should be designed to improve effectiveness. To achieve these aims, this study systematically reviewed and coded UB interventions in healthcare to align them with the BCTO [9] using directed content analysis based on study reports.

### Research questions

1. Which BCTs have been used in interventions to reduce UB between healthcare staff?
2. Which BCTs have not yet been used in interventions to reduce UB between healthcare staff, but could plausibly work?
3. How are BCTs used across intervention types?
4. Is there evidence that some BCTs are more effective than others in reducing UB?

## Methods

This study reports a rigorously undertaken review building on an initial realist review and adheres to PRISMA guidelines where possible [20].

### Systematic search

A systematic search was performed to update our realist review [15] to include intervention reports published up until 3 July 2024. Originally, we searched Embase, CINAHL and MEDLINE databases and for grey literature on HMIC, NICE Evidence Search, Patient Safety Network, Google and Google Scholar databases, and NHS Employers and NHS Health Education England websites. This update included MEDLINE, Embase, and CINAHL databases as well as the top 20 entries on Google Scholar. Google Scholar was searched using Harzing’s Publish or Perish software (https://harzing.com/resources/publish-or-perish). Grey literature was not searched for this update.

Full systematic search syntax is available in Supplementary File S1.

### Article selection

Screening was conducted in Rayyan.ai (www.rayyan.ai) by two independent reviewers (J.A. and B.U.). Any disagreements were resolved by discussion with a third reviewer.

Inclusion criteria were as follows (Table 1):

**Table 1:** Inclusion criteria

Category	Criterion
Study design	Any (including nonempirical papers/reports)
Study setting	Acute healthcare settings—acute, critical, emergency. Interventions could be delivered globally
Types of unprofessional behavior	All as exhibited and experienced between healthcare staff (not patients nor patient to staff)
Types of participants	Employed staff groups including students on placements
Types of interventions	Individual, team, organizational and policy level interventions. Cyber-bullying and other forms of online staff-to-staff unprofessional behavior
Outcomes	Included but not limited to a focus on one or more of: staff wellbeing (stress, burnout, resilience) staff turnover, absenteeism, malpractice claims, patient complaints, recruitment, patient safety (avoidable harm, errors, speaking up rates, safety incidents, improved listening/response), cost
Language	English only

### Data extraction

Characteristics of interventions were extracted from included studies for the purposes of understanding samples, intervention types, and more. Supplementary File S2 reports full study characteristics.

For each study, we extracted the written portion which described the intervention (usually in the methods or introduction sections of study reports or supplementary files) into a large table in Microsoft Word. This table had four main columns: source, intervention, intervention description, and BCT codes, similar to the officially published BCTO data extraction template [21]. This table was then used in the BCT coding process described below.

### BCT framework selection

As mentioned earlier, there are several BCT taxonomies/ontologies reported in the literature [9, 10, 22]. The most widely used and validated in health-related research settings is the BCTTv1 [8]—and this has been used in most existing efforts to analyses BCTs in literature syntheses [14, 22, 23]. However, as of 2024, the BCTTv1 has been recently revised and expanded to become the BCTO. This increased it from containing 93 hierarchically clustered techniques with the BCTTv1 to 284 in the BCTO. The prior BCTTv1 was primarily designed for health-related interventions (such as smoking, exercise, or obesity) rather than interventions that target nonhealth-related behavior. Due to its expansion, the BCTO has more widely applicable BCTs outside of just health related research, and is intended to be a “live” resource with continual updates in the future, suitable also for reading by “artificial intelligence agents” [9]. Because the BCTO has a focus on machine readability and alignment with standardized ontology repositories such as the ontology lookup service [9], we expect using the BCTO will ensure greater relevance of this review well into the future. To test the potential greater applicability of the BCTO to health services research, while considering it will likely be more forward compatible, we opted to use the BCTO rather than BCTTv1.

### Describing the BCTO

**Figure 1:**
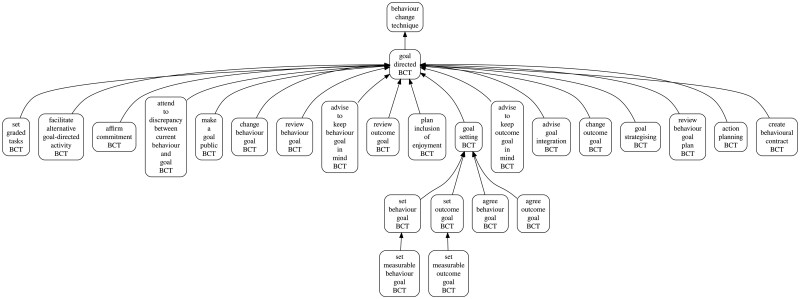
Example portion of the hierarchical nature of the BCT ontology for the parent class “goal-directed BCT”. Generated at https://bciovis.hbcptools.org/ and reproduced under CC-BY 4.0 license.

The BCTO is hierarchically ordered, as such, many “children” BCTs are contained within 20 higher-order parent classes of BCTs across five total possible hierarchical levels (Fig. 1). For example, Fig. 1 shows the portion of the BCTO oriented around the “goal-directed BCT.” Thus, “goal-directed BCT” is shown at the top as the parent class and this also has several “children” at multiple levels, for example “goal setting BCT” (which also has its own further children). If you add the total number of BCTs in the whole BCTO hierarchy together, there are 284 BCTs. Table 2 describes the 20 parent BCTs, how they are defined, and how many BCTs fall within the parent group. Although there are 284 total BCTs, to simplify the analysis and to make presentation of results possible, we collapsed the 284 total BCTs into their 20 parent classes.

**Table 2:** Overview of the BCT ontology and the 20 parent BCTs

BCT group	Definition	No. of BCTs in this group (as of December 2024) in BCTO
Goal-directed BCT	A behavior change technique that sets or changes goals.	25
Monitoring BCT	A BCT that involves gathering or using information about performance.	12
Social support BCT	A BCT that involves taking steps to secure or deliver the support or aid of another person.	16
Guide how to perform behavior BCT	A BCT that provides guidance regarding how to perform the behavior.	6
Conduct a behavioral experiment BCT	A BCT that advises on how to identify and test hypotheses about the behavior, its causes and consequences.	1
Suggest different perspective on behavior BCT	A BCT that suggests the deliberate adoption of a new perspective on the behavior.	5
Increase awareness of behavior BCT	A BCT that draws attention to the behavior.	3
Increase awareness of consequences BCT	A BCT that draws attention to consequences of the behavior in the normal course of events.	22
Awareness of other people’s thoughts, feelings or actions BCT	A behavior change technique that increases awareness of what other people think, do, or feel.	7
Associative learning BCT	A behavior change technique that involves repeated pairing of a stimulus with another stimulus or with a behavioral outcome.	15
Advise specific behavior BCT	A behavior change technique that advises the person to perform a behavior in a particular way to help change the target behavior.	9
Manage mental processes BCT	A behavior change technique that advises how to manage mental processes to facilitate the target behavior.	4
Prompt thinking related to successful performance BCT	A behavior change technique that prompts thinking relating to successful performance of a behavior.	6
Change the body BCT	A behavior change technique that alters the structure or functioning of the person’s body.	1
Promote pharmacological support BCT	A behavior change technique promoting medicines or other drugs.	3
Advise how to change emotions BCT	A BCT that suggests a method to alter emotions.	20
Restructure the environment BCT	A behavior change technique that alters the environment in which the behavior is, or would have been, performed in a way that facilitates or impedes the behavior.	12
Prompt focus on self-identity BCT	A BCT that prompts the person to focus on their mental representation of themselves.	5
Behavioral consequence BCT	A behavior change technique that alters the consequences or promised consequences for a behavior.	77
Outcome consequence BCT	A behavior change technique that alters the consequences or promised consequences for an outcome that results from performing or not performing a behavior.	35
Total	n/a	284

Reproduced and updated from Marques *et al.* under CC-BY license [9]

## Data Analysis

### Coding process

To code BCTs used in interventions, we used a directed content analysis approach [24] in line with other studies seeking to understand BCT usage [23, 25]. Two researchers (J.A. and B.U.), trained in use of the BCTO and BCTT based on available training resources (https://www.bciontology.org/module-1), read and independently coded the excerpts of each study line by line against the BCTO Revision 4 (May 2024) into the data extraction template. Coding was performed in July and August 2024. BCTs and coded passages were both color-coded according to BCT use based on intervention descriptions, and to ensure later clarity and ability to trace where BCT codes originated. Any coding differences were resolved through discussion between J.A. and B.U., without the need to involve the third author (J.M.).

#### Coding assumptions

We made several main assumptions in line with prior BCTT/BCTO classification studies [14, 25, 26]. First, we assumed that BCTs operated by modifying individual healthcare staff behavior (i.e. to be less unprofessional). Second, in cases where we had limited information to make more specific codes, we opted to code the less-specific parents of BCTs (e.g. “guide how to perform behavior BCT,” rather than “demonstrate the behavior BCT”). Third, we did not intend to code BCTs used in comparison/control arms of studies because our prior realist review found that it is extremely unlikely that any studies had a control arm, and that if they did, it would not contain relevant BCTs. Fourth, we identified two parent codes relating to consequences of BCTs (these are “outcome consequence BCT” and “behavioral consequence BCT”). Our analysis stuck mainly to the latter, because UB is what is attempted to be dissuaded in these interventions (rather than any outcomes of UB).

### Analyses conducted

To understand differences across intervention types, we broke down BCT usage according to the five categories of intervention we identified in our prior realist review [2]: single-session, multisession, combined session with other activity, professional accountability, and structured culture change interventions (Table 3). This classification process was performed based on intervention characteristics (e.g. duration, contact points, and intervention components such as reporting systems) in a customized Microsoft Excel spreadsheet to minimize risk of error.

**Table 3:** Intervention types and descriptors

Intervention type	Intervention design and content
Single-session interventions (*n* = 15)	Single, one-off lectures or workshops to try to change participant behavior. This can employ awareness-raising strategies such as education about UB, or can be combined with role-playing and other activities intended to enhance the ability to speak up and challenge UB in the moment.
Multisession interventions (*n* = 12)	These are similar to single-session interventions but rely on use of multiple workshops or lecture-type sessions over time. Most still draw on education and role-playing type activities.
Combined sessions with other activity interventions (*n* = 6)	These typically draw on single or multiple sessions as above, but also enhance this with nonsession-based activities such as implementing an organization-wide code of conduct.
Professional accountability interventions (*n* = 10 studies, *n* = 7 interventions)	These are more complex than those outlined above, relying on a reporting and escalation system. Examples include Ethos and Vanderbilt interventions. These interventions typically combined a reporting system with, in the case of Ethos, training to enhance speaking up and role-modelling by leadership or, in the case of Vanderbilt interventions, incorporated championing (i.e. encouraging individuals to role-model and espouse the benefits of the intervention).
Structured culture change interventions (*n* = 4)	These include CREW which offers a flexible package enabling organizations to respond to UB as needed, building upon (i) ongoing action planning to assess which strategies to implement and (ii) surveys to understand prevalence and spread of UB. Strategies included training on assertiveness, communication and conflict resolution, as well as management training for leaders and other strategies that help build rapport between staff.

Reproduced with permission from Maben *et al.* [2].

We calculated descriptive statistics including the frequency of use of BCTs across included studies, as well as analyzing which BCTs were not used. Box plots were generated to depict differences in frequency of BCTs across intervention types. Stata 18 was used for box plots and Excel was used for descriptive statistics such as medians and interquartile ranges. We explored the possibility of further statistical analysis of results with a statistician (e.g. chi-square tests of differences in BCT use between intervention types), however, it was determined that statistical significance testing was of limited additional utility if trends in the data were clear.

We also originally sought to calculate an “effectiveness index” or similar comparative measure of degree of effectiveness (to address research question 4). However, we determined this would not be possible as very few interventions reported being unsuccessful on their main outcome measure.

## Results

### Study selection

**Figure 2:**
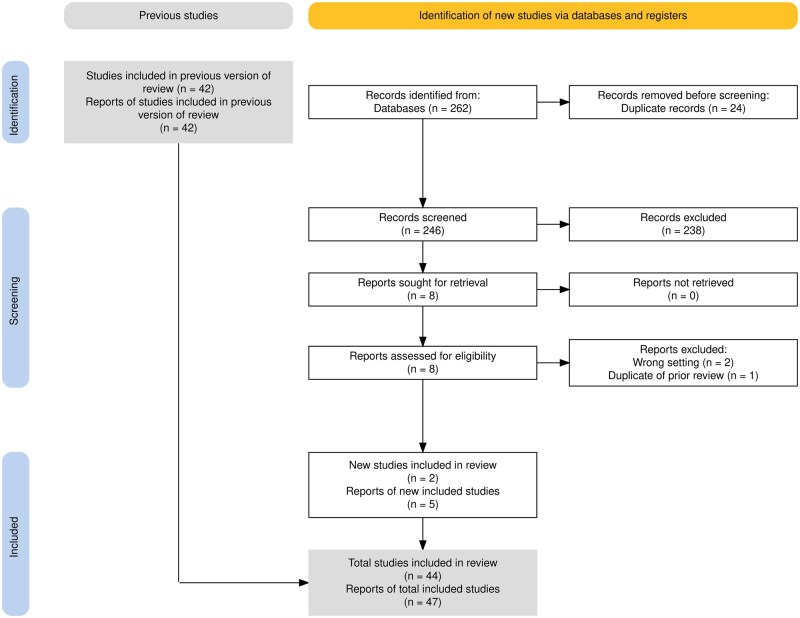
PRISMA diagram [20].

We initially included the 42 prior papers from our realist review (Fig. 2). From our search update, we identified 262 total papers from our new searches, which reduced to 246 after deduplication. We screened these 246 titles and abstracts, and excluded 238 due to lack of relevance to the topic. These eight full texts were then screened. Of these, five new reports were included [27–31]. One prior paper was also updated from a preprint version sent by a colleague to the published version [32]. This meant 47 reports were included [27–73].

### Number of interventions

We identified that four papers reported on multiple results of the same Ethos intervention taking place at the same time and in the same place [29, 31, 32, 36]. For our analysis of intervention content, these were combined into one and the BCTs were deduplicated across them. This meant that in total *n* = 44 interventions were included in our analysis of BCTs from the 47 included reports. Others used the same intervention [e.g. civility, respect, and engagement in the workforce (CREW)], but with modifications and delivered in different centers. Therefore, we considered them separate studies and did not combine them. Of the interventions, 31 were conducted in the United States [27, 33–35, 37–44, 46, 47, 50, 52, 56–65, 67–70, 72, 73], four in Australia [32, 45, 55, 66], three in Canada [28, 53, 54] two in South Korea [48, 49], and one in Turkey [71], Ireland [59], Greece [30], and Iran [51]. We have categorized these into the five intervention types from our previous work [2] (see Table 3).

### Study and intervention descriptions

Supplementary File S2 reports full intervention characteristics and BCTs used in each. Table 4 reports key information about interventions and number of BCTs used.

**Table 4:** Studies, BCTs used, and intervention type

Study	Sample	Intervention duration	Intervention description	Number of BCTs used	Parent class BCTs used (frequency)
**Single session interventions**					
Ceravolo *et al.* [34]	4032 practicing nurses, 1160 students and faculty	60–90-minute workshops over 3 years	Culture-change and communication enhancing workshops to decrease lateral violence in a five-hospital integrated health and care system	10	Social support BCT (2) Guide how to perform behavior BCT (2) Increase awareness of behavior BCT (1) Awareness of others’ thoughts, feelings, and actions BCT (1) Advise specific behavior BCT (1) Prompt focus on self-identity BCT (1) Behavioral consequence BCT (1) Increase awareness of consequences BCT (1)
Clark *et al.* [37]	65 senior nursing students	70-minute session	Study of an intervention which used problem based learning to address incivility	5	Increase awareness of consequences BCT (2) Increase awareness of behavior BCT (1) Guide how to perform behavior BCT (1) Monitoring BCT (1)
Dahlby and Herrick [38]	25 nurses on two nursing units	1.5-hour session	A 1.5 hour educational intervention focused on lateral violence, aiming to raise awareness and improve knowledge of causes	3	Increase awareness of behavior BCT (1) Guide how to perform behavior BCT (1) Prompt thinking related to successful performance BCT (1)
Duchesne *et al.* [28]	10 emergency medicine residents	One simulation session	Simulation to address microaggressions including cases of inferiority, sexual objectification, and invisibility	10	Guide how to perform behavior BCT (3) Advise specific behavior BCT (2) Increase awareness of behavior BCT (1) Awareness of others’ thoughts, feelings, and actions BCT (1) Social support BCT (1) Behavioral consequence BCT (1) Increase awareness of consequences BCT (1)
Embree *et al.* (2013) [42]	143 nurses	Two-hour session	Cognitive rehearsal education intervention on nurse to nurse lateral violence	11	Suggest different perspective on behavior BCT (3) Awareness of others’ thoughts, feelings, and actions BCT (2) Prompt focus on self-identity BCT (1) Prompt thinking related to successful performance BCT (1) Goal-directed BCT (1) Monitoring BCT (1) Associative learning BCT (1) Increase awareness of behavior BCT (1)
Griffin [43]	26 newly enrolled nurses	Two-hour session	Cognitive rehearsal to improve speaking up and intercolleague support to address lateral violence in newly licensed nurses	8	Increase awareness of consequences BCT (2) Increase awareness of behavior BCT (1) Guide how to perform behavior BCT (1) Social support BCT (1) Prompt thinking related to successful performance BCT (1) Monitoring BCT (1) Associative learning BCT (1)
Griffith *et al.* [44]	25 participants	One session of unknown length	An educational advance programme to aid residents and faculty in understanding and improving their learning environment. Attendees proposed coaching, signage, zero tolerance policies, and more, to tackle mistreatment	7	Goal-directed BCT (3) Awareness of others’ thoughts, feelings, and actions BCT (2) Increase awareness of behavior BCT (1) Prompt thinking related to successful performance BCT (1)
Hawkins *et al.* [45]	111 nurses across 12 units in four acute care hospitals	One session	Aimed to promote respectful workplace behavior by improving communication and aid recognizing, managing and mitigating negative workplace behavior	8	Guide how to perform behavior BCT (2) Prompt focus on self-identity BCT (2) Increase awareness of behavior BCT (1) Advise specific behavior BCT (1) Social support BCT (1) Goal-directed BCT (1)
Kile *et al.* [50]	19 nurses	2-hour training session	Cognitive rehearsal and education to address nurse-to-nurse incivility by enhancing speaking up and willingness to intervene	8	Advise specific behavior BCT (2) Increase awareness of behavior BCT (1) Guide how to perform behavior BCT (1) Social support BCT (1) Prompt thinking related to successful performance BCT (1) Associative learning BCT (1) Increase awareness of consequences BCT (1)
Mundo *et al.* [27]	32 participants (24 completing)	Presession preparation, 10 minute simulation, 12 and 13 minute debriefs.	Clinical simulation based training to confront racism, discrimination, and microaggressions	8	Guide how to perform behavior BCT (2) Advise specific behavior BCT (2) Social support BCT (1) Monitoring BCT (1) Behavioral consequence BCT (1) Associative learning BCT (1)
Nikstaitis and Simko [57]	21 nurses	1-hour session	A 60 minute educational programme to raise awareness and enhance speaking up	7	Guide how to perform behavior BCT (2) Increase awareness of behavior BCT (1) Awareness of others’ thoughts, feelings, and actions BCT (1) Social support BCT (1) Monitoring BCT (1) Increase awareness of consequences BCT (1)
O’Connell *et al.* [58]	76 participants	2-hour session	An education, cognitive rehearsal, and role play intervention to reduce lateral violence in a medical military setting	9	Guide how to perform behavior BCT (2) Advise specific behavior BCT (2) Increase awareness of behavior BCT (1) Awareness of others’ thoughts, feelings, and actions BCT (1) Social support BCT (1) Prompt thinking related to successful performance BCT (1) Increase awareness of consequences BCT (1)
Stagg *et al.* [65]	10 nurses	2-hour session	A cognitive rehearsal programme intended to improve speaking up and intervention.	5	Increase awareness of behavior BCT (1) Prompt thinking related to successful performance BCT (1) Associative learning BCT (1) Suggest different perspective on behavior BCT (1) Increase awareness of consequences BCT (1)
Stagg *et al.* [64]	20 nurses	2-hour session	A cognitive rehearsal programme intended to improve speaking up and intervention	9	Advise specific behavior BCT (2) Increase awareness of behavior BCT (1) Awareness of others’ thoughts, feelings, and actions BCT (1) Guide how to perform behavior BCT (1) Prompt thinking related to successful performance BCT (1) Associative learning BCT (1) Suggest different perspective on behavior BCT (1) Increase awareness of consequences BCT (1)
Warrner *et al.* [68]	60-bed orthopedic inpatient unit incl. management	45-minute session	An intervention comprising awareness education, cognitive rehearsal to enhance speaking up, and which included management	9	Prompt thinking related to successful performance BCT (2) Advise how to change emotions BCT (2) Increase awareness of behavior BCT (1) Behavioral consequence BCT (1) Associative learning BCT (1) Suggest different perspective on behavior BCT (1) Increase awareness of consequences BCT (1)
**Multisession interventions**					
Argyriadis *et al.* [30]	14 nurses	1 session per day for 10 days	An assertiveness training intervention to enhance speaking up and intervening	6	Monitoring BCT (3) Advise how to change emotions BCT (2) Manage mental processes BCT (1)
Asi Karakaş and Okanli [71]	30 nurses	Eight 2–2.5-hour sessions	Assertiveness training to improve speaking up, stress management, and appropriate communication	10	Guide how to perform behavior BCT (2) Advise how to change emotions BCT (2) Advise specific behavior BCT (2) Awareness of others’ thoughts, feelings, and actions BCT (1) Social support BCT (1) Prompt focus on self-identity BCT (1) Prompt thinking related to successful performance BCT (1)
Banerjee *et al.* [73]	Division faculty members (*n* = 41) and pulmonary and critical care fellows (*n* = 12)	13 × 1-hour sessions over 1 year	An antiracism educational study	6	Awareness of others’ thoughts, feelings, and actions BCT (2) Increase awareness of behavior BCT (1) Guide how to perform behavior BCT (1) Suggest different perspective on behavior BCT (1) Increase awareness of consequences BCT (1)
Barrett *et al.* [33]	An inpatient unit, critical care unit, emergency department, and inpatient operating room 59 preintervention and 45 postintervention nurses	Two 2-hour group sessions	A teambuilding intervention to reduce lateral violence by enhancing intercolleague relations	11	Awareness of others’ thoughts, feelings, and actions BCT (3) Guide how to perform behavior BCT (2) Prompt focus on self-identity BCT (2) Social support BCT (1) Goal-directed BCT (1) Monitoring BCT (1) Increase awareness of consequences BCT (1)
Demarco *et al.* [39]	5 graduate nursing student participants	2 hours per week for 6 weeks	A writing group study which was intended to enhance coping with UB rather than reducing UB directly	8	Monitoring BCT (3) Guide how to perform behavior BCT (2) Awareness of others’ thoughts, feelings, and actions BCT (1) Social support BCT (1) Advise how to change emotions BCT (1)
Jenkins *et al.* [47]	10 student leaders	6-hour long sessions, monthly, for 6 months	A series of journal club meetings that also incorporated journaling	5	Guide how to perform behavior BCT (1) Advise specific behavior BCT (1) Social support BCT (1) Prompt focus on self-identity BCT (1) Monitoring BCT (1)
Kang *et al.* [49]	40 hospital nurses	20 hours over 10 sessions	A cognitive rehearsal programme to address workplace bullying that also included role-playing and a focus on communication methods	7	Guide how to perform behavior BCT (2) Advise specific behavior BCT (2) Awareness of others’ thoughts, feelings, and actions BCT (1) Monitoring BCT (1) Associative learning BCT (1)
Kousha *et al.* [51]	80 emergency nurses	Five 2-hour sessions over 3 weeks	A cognitive rehearsal, education and role playing intervention that tested education only versus education plus cognitive rehearsal	8	Guide how to perform behavior BCT (2) Advise specific behavior BCT (2) Increase awareness of behavior BCT (1) Awareness of others’ thoughts, feelings, and actions BCT (1) Prompt thinking related to successful performance BCT (1) Increase awareness of consequences BCT (1)
Lasater *et al.* [52]	94 nursing staff	One 1-hour session comprising presentation and discussion on incivility, a 4-hour session on norm-setting and action planning, and a 2-hour simulation role playing session	An educational intervention with elements of simulation and norm-setting intended to reduce incivility	16	Increase awareness of consequences BCT (3) Advise specific behavior BCT (3) Guide how to perform behavior BCT (2) Prompt focus on self-identity BCT (2) Increase awareness of behavior BCT (1) Awareness of others’ thoughts, feelings, and actions BCT (1) Social support BCT (1) Goal-directed BCT (1) Monitoring BCT (1) Associative learning BCT (1)
Nicotera *et al.* [56]	19 participants with 47 comparison sample	6x 90-minute sessions	Intervention seeking to reduce “structurational divergence” and workplace conflicts through education, management, and role playing	11	Suggest different perspective on behavior BCT (3) Guide how to perform behavior BCT (2) Increase awareness of behavior BCT (1) Awareness of others’ thoughts, feelings, and actions BCT (1) Advise specific behavior BCT (1) Prompt thinking related to successful performance BCT (1) Restructure the environment BCT (1) Advise how to change emotions BCT (1)
O’Keeffe *et al.* [59]	203 participants in surgery	1-day session with a 1-hour follow-up e-learning course	An intervention drawing on simulations, case studies, and reflection exercises to improve conflict management	12	Awareness of others’ thoughts, feelings, and actions BCT (4) Advise specific behavior BCT (2) Increase awareness of behavior BCT (1) Guide how to perform behavior BCT (1) Social support BCT (1) Prompt thinking related to successful performance BCT (1) Monitoring BCT (1) Advise how to change emotions BCT (1)
Saxton [62]	17 participants	Two-day programme	Aiming to improve communication skills to address disruptive physician behavior	7	Advise specific behavior BCT (2) Increase awareness of behavior BCT (1) Awareness of others’ thoughts, feelings, and actions BCT (1) Guide how to perform behavior BCT (1) Prompt focus on self-identity BCT (1) Outcome consequence BCT (1)
**Combined session interventions**					
Chipps and McRury [35]	16 staff members	3 months	An educational session combined with a code of conduct and communication training seeking to reduce workplace bullying	17	Social support BCT (3) Goal-directed BCT (3) Increase awareness of consequences BCT (3) Prompt focus on self-identity BCT (2) Increase awareness of behavior BCT (1) Guide how to perform behavior BCT (1) Monitoring BCT (1) Behavioral consequence BCT (1) Suggest different perspective on behavior BCT (1) Restructure the environment BCT (1)
Dimarino [40]	Unknown	Unknown—”on demand” sessions and code of conduct	A code of conduct and educational intervention seeking to reduce lateral violence	8	Social support BCT (3) Advise specific behavior BCT (1) Monitoring BCT (1) Behavioral consequence BCT (1) Increase awareness of consequences BCT (1) Outcome consequence BCT (1)
Kang and Jeong [48]	72 hospital nurses	Two-hour familiarity session followed by 8 weeks on-demand usage (smartphone based)	A smartphone-based cognitive rehearsal intervention to reduce bullying	6	Guide how to perform behavior BCT (1) Advise specific behavior BCT (1) Social support BCT (1) Prompt thinking related to successful performance BCT (1) Associative learning BCT (1) Advise how to change emotions BCT (1)
Parker *et al.* [61]	Unclear/organization-wide	One away day and subsequent ongoing efforts of unclear overall duration	Multiple interventions used concurrently to reduce horizontal violence, making use of education, conflict management and leadership training, cognitive rehearsal, and a code of conduct	16	Awareness of others’ thoughts, feelings, and actions BCT (2) Guide how to perform behavior BCT (2) Social support BCT (2) Goal-directed BCT (2) Advise how to change emotions BCT (2) Increase awareness of behavior BCT (1) Advise specific behavior BCT (1) Prompt thinking related to successful performance BCT (1) Monitoring BCT (1) Behavioral consequence BCT (1) Increase awareness of consequences BCT (1)
Stevens [66]	Unclear	Unclear	Antibullying intervention including workshops for education, policies, supervisor training, and more	10	Awareness of others’ thoughts, feelings, and actions BCT (2) Goal-directed BCT (2) Guide how to perform behavior BCT (1) Social support BCT (1) Prompt focus on self-identity BCT (1) Monitoring BCT (1) Behavioral consequence BCT (1) Suggest different perspective on behavior BCT (1)
Thorsness and Sayers [67]	Approximately 100 surgical staff	Unclear	Intervention comprising making action plans for different staff groups, codes of conduct, and cultural change efforts	14	Goal-directed BCT (4) Behavioral consequence BCT (2) Increase awareness of behavior BCT (1) Awareness of others’ thoughts, feelings, and actions BCT (1) Prompt focus on self-identity BCT (1) Prompt thinking related to successful performance BCT (1) Monitoring BCT (1) Suggest different perspective on behavior BCT (1) Restructure the environment BCT (1) Increase awareness of consequences BCT (1)
**Professional accountability interventions**					
Baldwin *et al.* [72]	Three academic medical centers	September 2019 to August 2021 (2 years)	Vanderbilt reporting and escalation system that includes informal and formal resolution mechanisms for reported behaviors. Also includes communication and leadership training, for example	15	Guide how to perform behavior BCT (3) Awareness of others’ thoughts, feelings, and actions BCT (2) Increase awareness of behavior BCT (1) Advise specific behavior BCT (2) Monitoring BCT (2) Behavioral consequence BCT (2) Increase awareness of consequences BCT (2) Prompt thinking related to successful performance BCT (1)
Dixon-Woods *et al.* [41]	Organization-wide at Johns Hopkins Medicine	Two-year period, 2014–16	Reporting system with formal investigation systems. Aimed to improve speaking up behaviors	11	Behavioral consequence BCT (3) Awareness of others’ thoughts, feelings, and actions BCT (2) Guide how to perform behavior BCT (1) Advise specific behavior BCT (1) Social support BCT (1) Prompt focus on self-identity BCT (1) Restructure the environment BCT (1) Increase awareness of consequences BCT (1)
Ethos Intervention [29, 31, 32, 36]	Eight hospitals	5 years	Ethos reporting system with peer messengers, informal resolution, formal investigation, training to enhance speaking up and role-modelling	27	Social support BCT (6) Behavioral consequence BCT (5) Increase awareness of consequences BCT (4) Monitoring BCT (3) Awareness of others’ thoughts, feelings, and actions BCT (2) Goal-directed BCT (2) Guide how to perform behavior BCT (2) Increase awareness of behavior BCT (1) Prompt thinking related to successful performance BCT (1) Prompt focus on self-identity BCT (1)
Hickson *et al.* [46]	Unknown	Variable, depends on requirements	Vanderbilt graduated reporting intervention—also includes communication and leadership training, for example	16	Guide how to perform behavior BCT (3) Monitoring BCT (2) Behavioral consequence BCT (2) Prompt focus on self-identity BCT (2) Increase awareness of behavior BCT (1) Awareness of others’ thoughts, feelings, and actions BCT (1) Advise specific behavior BCT (1) Prompt thinking related to successful performance BCT (1) Goal-directed BCT (1) Increase awareness of consequences BCT (1) Outcome consequence BCT (1)
McKenzie *et al.* [55]	21 healthcare staff pre-to-post	18 months into a 3-year intervention	Vanderbilt approach using education, reporting systems with graduated intervention processes, safety champions, and action plans	9	Behavioral consequence BCT (3) Increase awareness of behavior BCT (1) Prompt focus on self-identity BCT (1) Goal-directed BCT (1) Monitoring BCT (1) Increase awareness of consequences BCT (1) Outcome consequence BCT (1)
Speck *et al.* [63]	Three teaching hospitals	4+ years	Variation on the Vanderbilt reporting system with graduated escalation from informal resolution (peer, then manager) to formal investigation, and championing. Chairs were able to report individuals to the committee rather than any staff member	8	Social support BCT (2) Behavioral consequence BCT (2) Awareness of others’ thoughts, feelings, and actions BCT (1) Prompt focus on self-identity BCT (1) Goal-directed BCT (1) Monitoring BCT (1)
Webb *et al.* [69]	Three hospitals	2 years for study data (but programme running for 9 years)	Vanderbilt reporting system with graduated escalation from informal resolution	13	Increase awareness of consequences BCT (3) Prompt focus on self-identity BCT (2) Monitoring BCT (2) Behavioral consequence BCT (2) Awareness of others’ thoughts, feelings, and actions BCT (1) Social support BCT (1) Prompt thinking related to successful performance BCT (1) Goal-directed BCT (1)
**Structured culture change interventions**					
Armstrong [70]	9 nurses	Two 8-hour sessions to train facilitators Four weeks total with one meeting per week. Sessions lasted 20–30 minutes	CREW intervention (using education, teambuilding exercises, roleplaying, and more)	11	Guide how to perform behavior BCT (3) Goal-directed BCT (2) Awareness of others’ thoughts, feelings, and actions BCT (2) Increase awareness of behavior BCT (1) Advise specific behavior BCT (1) Social support BCT (1) Suggest different perspective on behavior BCT (1)
Laschinger *et al.* [52]	8 units with 33 controls	6 months, selecting strategies from the CREW toolkit as appropriate	CREW intervention similar to above	7	Goal-directed BCT (3) Awareness of others’ thoughts, feelings, and actions BCT (2) Guide how to perform behavior BCT (1) Social support BCT (1)
Leiter *et al.* [54]	1173 workers across 41 units	6-months	CREW intervention similar to above	23	Goal-directed BCT (4) Awareness of others’ thoughts, feelings, and actions BCT (3) Guide how to perform behavior BCT (3) Social support BCT (3) Monitoring BCT (3) Behavioral consequence BCT (2) Increase awareness of behavior BCT (1) Advise specific behavior BCT (1) Prompt focus on self-identity BCT (1) Prompt thinking related to successful performance BCT (1) Suggest different perspective on behavior BCT (1)
Osatuke *et al.* [60]	647 postintervention CREW participants and 680 comparison (total 34 workgroups)	Flexible/various	CREW intervention (survey, action planning, various training e.g. on communication, education)	15	Social support BCT (4) Goal-directed BCT (4) Monitoring BCT (2) Awareness of others’ thoughts, feelings, and actions BCT (2) Increase awareness of behavior BCT (1) Guide how to perform behavior BCT (1) Suggest different perspective on behavior BCT (1)
Total	n/a	n/a		450	See Fig. 3 for total number of times BCT parent classes were used

### Which BCTs are used?

Across all studies, 477 individual uses of BCTs were coded. The median number of BCTs used per study was nine and the interquartile range was five. At a study level, the greatest number of BCTs used was by Leiter *et al.* [54] with 24 BCTs, and the smallest number was by Dahlby and Herrick [38] with three (Table 4). However, at an intervention level, with BCTs for the four Ethos study reports combined and deduplicated, we identified 27 BCTs used—making this intervention the most BCT-rich. This was also likely a function of collapsing four study reports into one [29, 31, 32, 36], increasing the total amount of intervention description we could draw on. At an intervention level, 450 total BCTs were used. The remainder of this paper reports intervention-level analysis.

### Frequency of BCT usage

**Figure 3:**
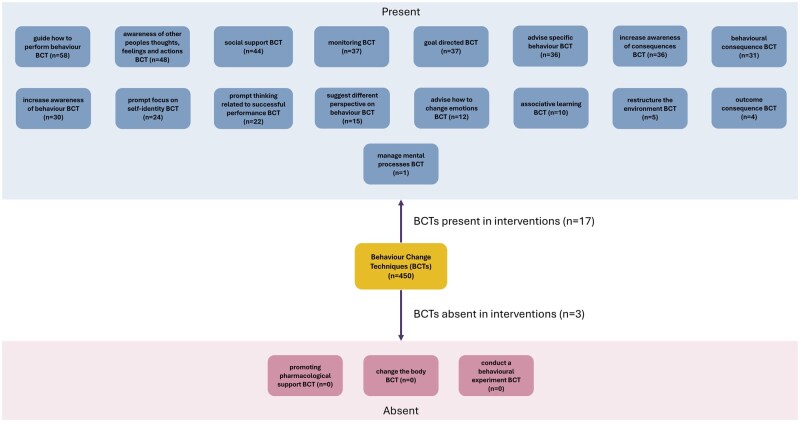
BCTs present and absent across interventions based on 20 parent BCT categories (number of times BCTs used within each category in parentheses).

Figure 3 shows presence and frequency of BCT use. The figure shows that only 17 out of 20 parent BCTs were represented across these interventions (and three were unused). The majority of interventions were either single-session or multisession types, and these take the form of workshops or lectures and are predominantly focused on educating staff about UB. Therefore, the most common BCTs encountered overall related to guiding how to perform behaviors (*n* = 58), and enhancing awareness of other peoples’ thoughts, feelings, and actions (*n* = 48). Social support BCTs (*n* = 44) were also highly common and were frequently used in interventions attempting to provide communication and assertiveness training. Professional accountability interventions rely more heavily on technology-based reporting systems. Therefore, these drew more heavily on “monitoring BCT” (*n* = 37) as they directly provide feedback about behaviors. These also used BCTs focused on provision of consequences for behaving poorly (*n* = 31) and improving awareness of these consequences (*n* = 36), which together are seemingly intended to deter future UB.

Several interventions [42, 43, 48–51, 58, 61, 64, 65, 68] also featured cognitive rehearsal, which often intended to enable staff to better speak up or intervene when UB is experienced. These interventions relied heavily on “prompt thinking related to successful performance BCTs” (*n* = 22) as they involve mentally practicing a behavior. Both cognitive rehearsal and general workshop or education-based interventions also could focus on improving speaking up as well as reducing UB and thus could recommend engaging in specific speaking up behaviors (advise specific behavior BCT, *n* = 36). “Prompt focus on self-identity BCT” (*n* = 24) was also used in more advanced intervention types, as this was associated with use of appointing champions or good-behavior role-models. Last, structured culture change systems were built around goal direct BCTs (*n* = 37) and providing social support.

Few interventions used “managing mental processes BCTs,” which largely relate to regulation of unconscious mental processes—this may reflect that most UBs are considered to be driven by conscious (not unconscious) decision-making. Few interventions attempted to directly restructure the physical or social environment (e.g. by putting up posters about UB or by rearranging how workers sit); rather, studies focused on indirectly increasing social support. Furthermore, few interventions tried to use BCTs relating to consequences of a behavior (e.g. for smoking this would be to highlight the damaging diseases that might arise from smoking)—rather, they targeted consequences of engaging in UB (e.g. that engaging in UB will directly result in negative social consequences).

Three of twenty BCT parent categories were absent: “changing the body BCT,” “promoting pharmacological support BCT” and “conducting a behavioral experiment BCT” (Fig. 3). This is not surprising as these BCTs are of more relevance to health promotion type interventions and are unlikely to be of significant benefit in UB-related interventions.

### Patterns of BCT use

**Figure 4:**
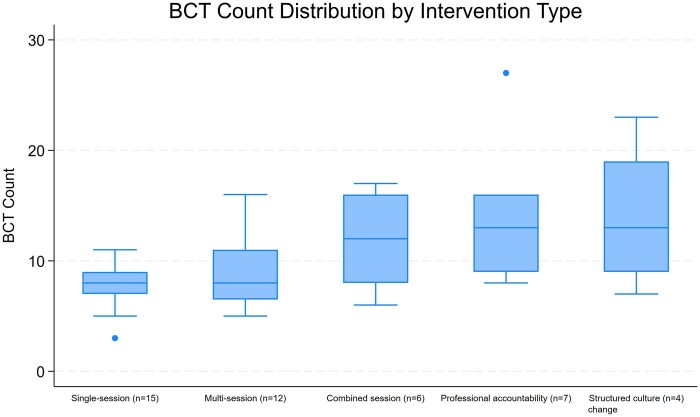
Distribution of BCT count across different intervention types. The box plot displays the median, interquartile range, and potential outliers for the frequency of BCT use in each study type.

Figure 4 depicts frequency of use of BCTs across different intervention types. It shows that as intervention complexity increases, so does the median frequency of use of BCTs across these interventions.

#### Differences in BCT use across intervention types

Table 5 shows the proportion of BCTs used within each intervention type, that is that 23.2% of BCTs used by structured culture change interventions were “goal-directed BCTs.” Supplementary File S3 also shows the same information in a graphical format. This allows for comparison of relative BCT use across intervention types.

**Table 5:** Differences in BCTs used across intervention types

Intervention type	Single-session (*n* = 15)	Multisession (*n* = 12)	Combined session (*n* = 6)	Professional accountability (*n* = 7)	Structured culture change (*n* = 4)	Total BCT use (*n* = 44 interventions)
Guide how to perform behavior BCT	15.4% (18/117)	16.8% (18/107)	7% (5/71)	9.1% (9/99)	14.3% (8/56)	58
Awareness of other peoples’ thoughts, feelings and actions BCT	7.7% (9/117)	15% (16/107)	7% (5/71)	9.1% (9/99)	16.1% (9/56)	48
Social support BCT	7.7% (9/117)	5.6% (6/107)	14.1% (10/71)	10.1% (10/99)	16.1% (9/56)	44
Goal-directed BCT	4.3% (5/117)	1.9% (2/107)	15.5% (11/71)	6.1% (6/99)	23.2% (13/56)	37
Monitoring BCT	4.3% (5/117)	10.3% (11/107)	7% (5/71)	11.1% (11/99)	8.9% (5/56)	37
Advise specific behavior BCT	10.3% (12/117)	14% (15/107)	4.2% (3/71)	4% (4/99)	3.6% (2/56)	36
Increase awareness of consequences BCT	10.3% (12/117)	5.6% (6/107)	8.5% (6/71)	12.1% (12/99)	0% (0/56)	36
Behavioral consequence BCT	3.4% (4/117)	0% (0/107)	8.5% (6/71)	19.2% (19/99)	3.6% (2/56)	31
Increase awareness of behavior BCT	12% (14/117)	5.6% (6/107)	4.2% (3/71)	4% (4/99)	5.4% (3/56)	30
Prompt focus on self-identity BCT	3.4% (4/117)	6.5% (7/107)	5.6% (4/71)	8.1% (8/99)	1.8% (1/56)	24
Prompt thinking related to successful performance BCT	8.5% (10/117)	3.7% (4/107)	4.2% (3/71)	4% (4/99)	1.8% (1/56)	22
Suggest different perspective on behavior BCT	5.1% (6/117)	3.7% (4/107)	4.2% (3/71)	0% (0/99)	3.6% (2/56)	15
Advise how to change emotions BCT	1.7% (2/117)	6.5% (7/107)	4.2% (3/71)	0% (0/99)	0% (0/56)	12
Associative learning BCT	6% (7/117)	1.9% (2/107)	1.4% (1/71)	0% (0/99)	0% (0/56)	10
Restructure the environment BCT	0% (0/117)	0.9% (1/107)	2.8% (2/71)	1% (1/99)	1.8% (1/56)	5
Outcome consequence BCT	0% (0/117)	0.9% (1/107)	1.4% (1/71)	2% (2/99)	0% (0/56)	4
Manage mental processes BCT	0% (0/117)	0.9% (1/107)	0% (0/71)	0% (0/99)	0% (0/56)	1
Total times BCTs used in intervention type	117 (100%)	107 (100%)	71 (100%)	99 (100%)	56 (100%)	450
Median number of BCTs used in intervention type (IQR)	8 (7–9)	8 (6.75–15)	12 (8.5–15.5)	13 (10–15.5)	13 (10–17)	9 (7–11.25)

Total times BCTs were coded for each intervention type are in brackets (e.g. single-session interventions used 117 BCTs in total).

### BCTs and effectiveness

Unfortunately, it was not possible to improve understanding of which BCTs were most effective. While 30 out of 44 interventions were evaluated in some form in this literature, almost all studies reported having some level of positive effect. Out of these 30, three reported no positive results [42, 58, 74] and three had negative results [35, 51, 57]. However, in these negative result studies, their primary aim was to reduce reports of UB by staff, but they found more reports of UB after the intervention when compared to baseline. In contrast to the authors of those studies, we would not necessarily consider this to indicate a lack of effectiveness, because a short-term increase in reports of UB would logically be expected to occur as a result of increased awareness of UB and/or increases in speaking up. A drop in UB reports (reflecting an actual decrease in “real” UB) may only occur after a very long period of time (e.g. 5 years as seen in the Ethos intervention [32]). Better use of logic models in design of these interventions might help to avoid such misinterpretations from occurring. As such, due to these issues, there was not the sensitivity required to be able to calculate an “effectiveness index” or similar.

## Discussion

Many interventions in health services that seek to change behavior of healthcare staff are often developed and championed by a few impassioned individuals working within healthcare organizations and, understandably, not all may be trained in use of implementation or behavioral science. This research sought to understand use of BCTs in these types of interventions by coding 44 unique interventions to reduce UB between healthcare staff against the BCTO. Therefore, this is the first study to show that the BCTO is applicable in health services research [9]. This analysis also allowed us to demonstrate that multicomponent interventions for UB reduction could benefit from drawing on behavioral science frameworks to improve replicability, understanding of what might drive effectiveness, and transparency of their reporting. We found that the most frequent BCTs used in these 44 interventions related to providing education (e.g. “awareness of behavior BCT,” and “guide how to perform behavior BCT”). This is not surprising, as almost all interventions had some component of education about what UBs look like and provision of encouragement and education on how to speak up.

Interventions featured different patterns of BCTs. Session-based interventions had greater focus on education and role playing. More advanced intervention types such as professional accountability (e.g. Ethos [32]) and structured culture change interventions (e.g. CREW [54, 60]) had different patterns of BCT use. Professional accountability interventions often draw on technological reporting systems and this enabled these to more heavily utilize BCTs which both provide and promise greater consequences for instigators if they engage in UBs. Structured culture change interventions revolve more around providing space and time for staff to set goals to tackle issues encountered on an ad hoc basis and this is reflected in the goal setting BCTs used.

Due to few studies formally evaluating these interventions, and few studies that reported any negative results, creation of an “effectiveness index” or similar [22] to understand which BCTs may be most effective, was not possible. However, we found that more complex interventions with more rigorous evaluations drew more frequently on consequence-related BCTs, increasing social support, and goal-directed BCTs (e.g. goal setting). More evaluations of robustly reported interventions are needed to gain further understanding of which BCT choices may enhance intervention effectiveness.

### BCTs that could plausibly work in UB interventions

Some BCTs were not used by interventions analysed here, but should still be tested in future interventions to address UB. Key examples include “making a goal public BCT” (which may plausibly increase social pressure to behave professionally), “restructuring of the physical environment” (e.g. providing more spaces to relax during the workday), and “action planning” (e.g. creating specific goals around when and how to speak up if UB is encountered). This maps with our realist review findings, which suggested healthcare organizations over focus on individual behavior change rather than organizational-level culture change [15]. Only structured culture change interventions made much use of goal-directed BCTs overall. There were also relatively few reward-related BCTs coded, indicating interventions relied more on providing negative consequences rather than positive ones. Only certain professional accountability interventions such as Ethos enable reporting of “positive” behaviors [32]; but it is unclear whether encouraging positive behavior reduces likelihood of also engaging in negative behavior in other circumstances. Enabling reporting of positive behaviors may nonetheless reduce the potential negative impact of these systems on staff wellbeing and wider organizational culture [2, 7].

### Applicability of the BCTO to organizational interventions

While the BCTO proved generally suitable for this research area, challenges were encountered. This included difficulties in distinguishing between closely related BCTs, and the extensive coding required by the expanded BCTO with 284 BCTs. The BCTO shows practical examples of how BCTs are described in the online tool (available at https://www.bciosearch.org/), but more examples of each BCT from more than one field would better enable objectivity of coding across studies and reviewers. However, provision of resources such as free online training, and the ability to search the ontology, aided our analysis process. We also found it difficult to find BCT analogues for certain situations. For example, professional accountability interventions have an escalating pyramid of accountability which makes potential instigators aware of worsening consequences for engaging in poor behavior repeatedly—this may be analogous to a strengthening “dose” of particular BCTs [75].

The BCTO may also benefit from inclusion of organizational-level techniques to fully capture the complexity of behavior change interventions in healthcare settings. We have previously advised that many interventions are highly individualized and that intervention architects should, where possible, focus more on taking accountability for historically poor organizational cultures, redesigning workplace processes to avoid role overlaps and conflict, increasing resources and/or staffing to reduce stress on employees, and improving management skills, among others [7]. Many of these organizational-level actions do not have good analogues in the BCTO, as the BCTO is still individual behavior-focused. Future revisions of the BCTO may want to bring in other organizational-level or workplace-specific BCTs to address this limitation. However, this did not significantly limit this review, as, currently, few interventions try these organizational and system-level changes to tackle UB as they are quite difficult to implement (e.g. only one out of 42 interventions in our prior review attempted workplace redesign of any kind) [2].

### Strengths and limitations

This study has several strengths; it involved a rigorous and time-intensive coding process against the largest current typology/ontology of BCTs available with independent screening throughout, and wide intervention inclusion criteria. To date, we have not seen published examples of studies attempting coding against the BCTO (only the prior taxonomy [8]). Our finding that use of BCTs is different across intervention types validates the utility of our prior intervention classification, which reinforces the validity of our prior work based on this additional understanding of intervention components [76].

This study also has several limitations. One limitation was that this analysis is still only as good as reporting of interventions themselves. As such, due to poor reporting, it is likely that some BCTs were either misinterpreted or unable to be coded. Many of these interventions would likely be considered poor quality by a formal systematic review, and publication bias may have affected which interventions we were able to synthesize. Some aspects of coding are inevitably subject to a degree of subjectivity. Lastly, we did not analyses MoAs or modes of delivery of BCTs. This is due mainly to lack of published guidance about which BCTs are evidenced to match with particular MoAs at the time the work was conducted, as well as due to poor reporting by included studies.

## Conclusion

This study is the first to align 44 unique interventions aimed at reducing UB among healthcare staff with the behavior change technique ontology (BCTO) and, to our knowledge, is the first synthesis to align any interventions with the new BCTO. In doing so, we showed that aligning health services behavior change interventions with behavioral science frameworks is possible, and using such frameworks should be encouraged when designing future interventions targeting healthcare staff behaviour. We identified that more complex interventions tend to incorporate more total BCTs. By categorizing these BCTs into 20 parent classes, of which 17 had representation in the included studies, we found that educational components—specifically those increasing awareness of UB and guiding individuals on addressing such behaviors—were the most frequently employed techniques. Additionally, distinct patterns emerged where session-based interventions focused on education and role-playing, whereas more advanced interventions—such as professional accountability and structured culture change interventions—utilized consequence-related BCTs, enhanced social support mechanisms, and goal-directed strategies such as goal setting. This suggests that a broader range of BCTs may contribute to the effectiveness of more complex interventions. Future research on UB in healthcare should focus on more robust evaluations of complex interventions with proper reporting to determine what BCT combinations are most effective.

## Supplementary Material

ibaf058_Supplementary_Data

## Data Availability

De-identified data from this study are not available in a public archive. De-identified data from this study will be made available (as allowable according to institutional ethical standards) by emailing the corresponding author.
