# Supplementary material for: Systematically analyzing behavior change techniques used in 44 interventions to reduce unprofessional behavior between healthcare staff
Source: Transl Behav Med. 2025 Oct 15;15(1):ibaf058. doi: 10.1093/tbm/ibaf058 (PMC12527449; doi:10.1093/tbm/ibaf058)
Supplement: ibaf058_Supplementary_Data [file ibaf058_supplementary_data.zip › Supplementary File 2 - Full table.docx]

# Intervention characteristics and BCTs used within them

| **Study** | **Sample*** | **Duration** | **Design and data collection timepoints** | **Theoretical framework** | **Intervention strategies** | **Measures** | **Findings** | **BCTs** |
| --- | --- | --- | --- | --- | --- | --- | --- | --- |
| ***Single-session interventions*** | | | | | | | | |
| Ceravolo et al. (2012)(32) | 4032 practicing nurses, 1160 students and faculty | 60-90 minute workshops over 3 years | Quasi-experimental pre-post design without control. Pre (2007) and post 3-year data collection via survey (2011). | None | Education / awareness (assertiveness training), role modelling | Verbal Abuse Survey | This intervention used culture-change and communication enhancing workshops to decrease lateral violence in a five-hospital integrated health and care system. Nursing turnover and vacancy rates decreased but it was not clear if that was due to the intervention. | identify self as role model BCT  BCIO:007158  inform about negative emotional consequences BCT  BCIO:007177  increase salience of behaviour BCT BCIO:007175  instruct how to perform a behaviour BCT BCIO:007058  deliver informational support BCT  BCIO:007042  practice behaviours  BCIO:007094  demonstrate the behaviour BCT  BCIO:007055  present information from credible influence BCT  BCIO:007075  promise positive consequence for alternative behaviour BCT BCIO:007204  arrange informational support BCIO:007037 |
| Clark, Ahten and Macy (2013)(35) | 65 senior nursing students | 70 minute session | Pre-post design without control.  Only post-measures were delivered, immediately post-session. | Unknown | Problem-based learning, role playing | Custom feasibility questionnaire | Study of an intervention which used PBL to address incivility. It was found to heighten awareness of incivility, improve favourable reaction to PBL, and give increased confidence. | prompt comparative imagining of future outcomes BCT  BCIO:007070  increase awareness of consequences BCT  BCIO:007062  demonstrate the behaviour BCT  BCIO:007055  observe behaviour without feedback BCT  BCIO:007018  increase awareness of behaviour BCT BCIO:007173 |
| Dahlby and Herrick (2014)(36) | 25 nurses on two nursing units. | 1.5 hour session | Pre-post design without control. Posttest timing is unclear. | None | Education, cognitive rehearsal | Lateral and Vertical Violence in Nursing Survey | A study examining the effects of a 1.5 hour educational intervention on lateral violence. They found participants were better able to identify causes of lateral violence and it perhaps contributed to reducing frequency too. However, no statistically significant results were noted other than for lateral violence becoming less serious post-intervention. | prompt mental rehearsal of successful performance BCT BCIO:007138  instruct how to perform a behaviour BCT  BCIO:007058  increase awareness of behaviour BCT  BCIO:007173 |
| Duchesne et al. (2023) (22) | 10 emergency medicine residents | One simulation session | Post-only pilot study. | Kern's six-step model | Simulation-based learning, reflection, awareness raising, allyship, provision of resources | Qualitative feedback on the session, key takeaways regarding awareness of microaggressions, skills for allyship, and the use of specific toolkits to address microaggressions, gathered through an anonymous survey after the simulation | Increased awareness of gender-related microaggressions, gained practical strategies for allyship, and appreciated the safe space for reflection. Participants highlighted the usefulness of specific response toolkits and expressed interest in future sessions on broader microaggressions​ | remove aversive social consequence for behaviour BCT BCIO:007290  awareness of other peoples thoughts, feelings and actions BCT  BCIO:007072  context-specific repetition of alternative behaviour BCT  BCIO:007097  demonstrate the behaviour BCT  BCIO:007055  guide how to perform behaviour BCT BCIO:007050  increase awareness of behaviour BCT BCIO:007173  increase awareness of consequences BCT  BCIO:007062  instruct how to perform a behaviour BCT BCIO:007058  deliver appraisal support BCT BCIO:007043  practice behaviours  BCIO:007094 |
| Embree, Bruner and White (2013)(40) | 143 nurses | Two-hour session | Quasi-experimental pre-post design without control. Some post-measures were immediate and others such as internalised sexism were one-year later. | None | Education, cognitive rehearsal | Internalized Sexism, Minimization of Self, Total Nurse Workplace Behavior Scale, and Silencing the Self-Work Scale, RN voluntary turnover | Investigates effectiveness of a cognitive rehearsal education intervention on nurse to nurse lateral violence. It found that there was a trend towards increased awareness of lateral violence but no statistically significant results were identified. | awareness of other peoples thoughts, feelings and actions BCT  BCIO:007072  provide feedback on behaviour BCT  BCIO:007023  adopt positive self-identity BCT  BCIO:007161  inform about antecedents BCT BCIO:007052  present information from credible influence BCT  BCIO:007075  increase awareness of behaviour BCT BCIO:007173  draw attention to incompatible beliefs BCT  BCIO:007057  suggest different perspective on behaviour BCT  BCIO:007302  goal strategising BCT  BCIO:007008  cue BCT BCIO:007081  prompt mental rehearsal of successful performance BCT BCIO:007138 |
| Griffin (2004)(41) | 26 newly-enrolled nurses | Two-hour session | Quasi-experimental pre-post design without control. Data were collected post-only, occurring one-year post-intervention. | Cognitive learning theories | Cognitive rehearsal | Focus groups | Investigates a cognitive rehearsal intervention as a shield for lateral violence in newly licensed nurses. It finds that knowledge of lateral violence enabled greater coping and confronting skills. Retention rate improved. | prompt mental rehearsal of successful performance BCT BCIO:007138  cue BCT BCIO:007081  increase awareness of behaviour BCT BCIO:007173  deliver informational support BCT  BCIO:007042  instruct how to perform a behaviour BCT  BCIO:007058  increase awareness of consequences BCT  BCIO:007062  prompt comparative imagining of future outcomes BCT  BCIO:007070  provide feedback on outcome of behaviour BCT  BCIO:007027 |
| Griffith et al. (2019)(42) | 25 participants | One session of unknown length | Quasi-experimental post-only design without control. Data collection was only post-intervention via survey over an unclear timeline. | Six-step approach to curricular development by Kern et al. | Education, action planning, keeping records | Custom electronic evaluation of effectiveness | The authors developed an educational advance programme to aid residents and faculty in understanding and improving their learning environment. Attendees proposed coaching, signage, zero tolerance policies, and more, to tackle mistreatment. There was some evidence it increased awareness. | increase awareness of behaviour BCT BCIO:007173  awareness of other peoples thoughts, feelings and actions BCT  BCIO:007072  present information from credible influence BCT  BCIO:007075  prompt thinking related to successful performance BCT  BCIO:007239  action planning BCT  BCIO:007010  goal strategising BCT  BCIO:007008  goal setting BCT  BCIO:007002 |
| Hawkins et al. (2022)(43) | 111 nurses across 12 units in four acute care hospitals | One session | Quasi-experimental pre-post design with control group (non-randomised). Baseline data collection occurred over the month prior to the intervention and post-data collection occurred over 6 months. | None | Education | Negative Acts Questionnaire— Revised, Ways of Coping Questionnaire | The intervention group experienced less of a reduction in bullying and negative behaviours than the control group. But no results were statistically significant. Authors recommended against single-session attempts to reduce UB. | instruct how to perform a behaviour BCT BCIO:007058  practice behaviours  BCIO:007094  demonstrate the behaviour BCT  BCIO:007055  increase awareness of behaviour BCT BCIO:007173  deliver informational support BCT  BCIO:007042  prompt focus on self-identity BCT BCIO:007157  identify self as role model BCT BCIO:007158  goal strategising BCT  BCIO:007008 |
| Kile et al. (2019)(48) | 19 nurses | 2-hour training session | Pilot study with no control group with pre, post (immediate), and 6 week follow-up | Bandura’s social learning theory (1977) | Education, cognitive rehearsal | Adapted versions of the nursing Incivility Survey (NIS), the Nurse Interaction subscale of the National Database of Nursing Quality Indicators (NDNQI), and a questionnaire with two open‐ended questions | Investigated with a mixed methods pilot study the impact of incivility and cognitive rehearsal education on nurse-to-nurse incivility. They found that it was effective at increasing recognition and confronting of incivility due to movement in means on the NIS subscales, and reduced perceived incivility. | increase awareness of behaviour BCT BCIO:007173  deliver informational support BCT  BCIO:007042  prompt mental rehearsal of successful performance BCT BCIO:007138  context-specific repetition of alternative behaviour BCT  BCIO:007097  Cue BCT BCIO:007081  demonstrate the behaviour BCT  BCIO:007055  practice behaviours  BCIO:007094  increase awareness of consequences BCT  BCIO:007062 |
| Mundo et al. (2024) (21) | 32 participants (24 completing) | Pre-session preparation, 10 minute simulation, 12 and 13 minute debriefs. | Pre-post | Self-determination theory and experiential learning | Role play, reflection, education | A survey with Likert-type scales assessing ability to identify racism, discrimination, and microaggressions (RDM), use upstander communication tools, and intervene in RDM situations. | After the workshop, there was a notable increase in self- perceived ability and confidence in identifying RDM (from 7 ± 3.2 to 8.6 ± 1.6, p < 0.003), using upstander communication tools (from 6.1 ± 3.5 to 8.5 ± 1, p < 0.0001), and the likelihood of intervening in RDM situations (from 7.1 ± 3.3 to 8.8 ± 1.1, p < 0.0002). | context-specific repetition of alternative behaviour BCT  BCIO:007097  demonstrate the behaviour BCT  BCIO:007055  practice behaviours  BCIO:007094  systematically desensitise BCT BCIO:007089  guide how to perform behaviour BCT BCIO:007050  remove aversive social consequence for behaviour BCT BCIO:007290  deliver appraisal support BCT BCIO:007043  provide feedback BCT  BCIO:007022 |
| Nikstatis and Simko (2014)(55) | 21 nurses | 1-hour session | Pilot study pre-post design without control group. Both pre- and post-data collection occurred over a 3-week timeframe. | None | Education, group discussion | Nursing incivility scale | A quantitative pilot study using a 1-group pre and post intervention test design to assess a 60 minute educational programme. They found that it increased perceived incivility. However this was not statistically significant. | monitoring BCT  BCIO:007017  increase awareness of consequences BCT  BCIO:007062  arrange informational support BCT BCIO:007037  suggest how to perform behaviour BCT BCIO:007303  increase awareness of behaviour BCT  BCIO:007173  awareness of other peoples thoughts, feelings and actions BCT  BCIO:007072  guide how to perform behaviour BCT  BCIO:007050 |
| O’Connell, Garbark and Nader (2019)(56) | 76 participants | 2-hour session | A pre-post study design with no control group. | None | Education, cognitive rehearsal, role play | Negative Acts Questionnaire - Revised (NAQ-R) | A quantitative exploration of nurses’ perceptions of lateral violence within a military setting and the impact of an education, cognitive rehearsal, and role play intervention. Six negative acts occurred daily or weekly preintervention and nine occurred postintervention. Putting together staff on different hierarchy levels into one session may have undermined results. | present information from credible influence BCT  BCIO:007075  increase awareness of behaviour BCT BCIO:007173  instruct how to perform a behaviour BCT  BCIO:007058  demonstrate the behaviour BCT  BCIO:007055  increase awareness of consequences BCT  BCIO:007062  deliver informational support BCT  BCIO:007042  context-specific repetition of alternative behaviour BCT  BCIO:007097  practice behaviours  BCIO:007094  prompt mental rehearsal of successful performance BCT BCIO:007138 |
| Stagg et al. (2017)(62) | 10 nurses | 2-hour session | Pilot study with post-only testing (6-months after the intervention over a 6-week period) without control. | None – based on Griffin (2004) | Education, cognitive rehearsal | Workplace Bullying Follow-Up Survey | Assessed the effectiveness of a 2-hour cognitive rehearsal programme, 6 months after completion. 70% of nurses reported changing behaviours, and 40% reported a decrease in bullying behaviours. | increase awareness of behaviour BCT BCIO:007173  cue BCT BCIO:007081  inform about antecedents BCT BCIO:007052  increase awareness of consequences BCT  BCIO:007062  prompt mental rehearsal of successful performance BCT BCIO:007138 |
| Stagg et al. (2011)(63) | 20 nurses | 2-hour session | Pre-post quasi-experimental design with no control group. Unclear when post-test was delivered. | None – based on Griffin (2004) | Education, cognitive rehearsal | Adapted Workplace Bullying Inventory | Evaluated a workplace bullying cognitive rehearsal programme. Significant differences were found in the results between pre-test and post-test for observed bullying (Z = -2.636, p < .01), bullying of others (Z = -2.449, p < .05), and sufficiency of the training on management of bullying (Z = -3.358, p < .01). | increase awareness of behaviour BCT BCIO:007173  cue BCT BCIO:007081  inform about antecedents BCT BCIO:007052  increase awareness of consequences BCT  BCIO:007062  practice behaviours  BCIO:007094  demonstrate the behaviour BCT  BCIO:007055  prompt mental rehearsal of successful performance BCT BCIO:007138  substitute behaviour BCT  BCIO:007095  suggest to change behaviour BCT  BCIO:007076 |
| Warrner et al. (2016)(66) | 60-bed orthopaedic inpatient unit incl. management | 45-minute session | Pre-post design without control group, with follow-up at 2-months post-intervention. | None | Education, cognitive rehearsal, environmental modification | Nursing Incivility Scale (NIS) | Evaluates an intervention comprising awareness education, cognitive rehearsal, and which included management. None of the scores for the five subscales of sources of incivility were statistically significant. Two out of five subscales showed a statistically significant decrease in instances of perceived incivility: general incivility (2.75 to 2.24, p = 0.00) and physician incivility (2.79 to 2.43, p = 0.04), and the others decreased but not significantly. | inform about antecedents BCT BCIO:007052  increase awareness of consequences BCT  BCIO:007062  increase awareness of behaviour BCT BCIO:007173  prompt thinking related to successful performance BCT BCIO:007239  advise behavioural ways to increase positive emotions BCT BCIO:050350  advise behavioural ways to reduce negative emotions BCIO:050333  promise positive consequence for alternative behaviour BCT BCIO:007204  cue BCT BCIO:007081  prompt mental rehearsal of successful performance BCT BCIO:007138 |
| ***Multi-session interventions*** | | | | | | | | |
| Argyriadis et al., (2023) (24) | 14 nurses | 1 session per day for 10 days | Experimental trial | None | Mindfulness based training | Blood pressure, heart rate, sleep quality, qualitative (stress levels, cognitive function) and observation (levels of conflict, healthcare practice, and emotionality) | The mindfulness intervention improved nurses' cognitive function (attention, memory), emotional regulation, and interpersonal relationships, reducing stress, tension, and anger. Nurses in the intervention group experienced better sleep quality (33.8% deep sleep vs. 29.1% in the control), lower heart rates (average 52 bpm vs. 61 bpm), and increased job satisfaction. | advise cognitive ways to increase positive emotions BCT  BCIO:050334  advise cognitive ways to reduce negative emotions BCT  BCIO:050337  self-monitor outcome of behaviour BCT BCIO:007025  monitor emotional consequences BCT BCIO:007066  enable person to manage automatic responses BCT BCIO:007143  provide biofeedback BCT  BCIO:007026 |
| Asi Karakaş and Okanli (2015)(28) | 30 nurses | Eight 2-2.5 hour sessions | Quasi-experimental pre-post design without control. Assessment was 6-months post-intervention. | None | Assertiveness training | Mobbing Scale, Rathus Assertiveness Inventory | This study reported an evaluation of an assertiveness training intervention in 30 nurses who experienced a high level of mobbing. Results indicated a statistically significant fall in mobbing after the intervention from 226.4 +- 27.7 to 159.6 +- 47.9 on the mobbing scale and an increase in assertiveness from 6.23 +- 15.6 to 17.0 +- 16.06. | context-specific repetition of alternative behaviour BCT  BCIO:007097  advise cognitive ways to increase positive emotions BCT  BCIO:050334  advise cognitive ways to reduce negative emotions BCT  BCIO:050337  awareness of other peoples thoughts, feelings and actions BCT  BCIO:007072  instruct how to perform a behaviour BCT BCIO:007058  persuade about personal capability BCT  BCIO:007137  adopt positive self-identity BCT  BCIO:007161  deliver appraisal support BCT BCIO:007043  demonstrate the behaviour BCT  BCIO:007055  practice behaviours  BCIO:007094 |
| Banerjee et al. (2022)(30) | Division faculty members (n = 41) and pulmonary and critical care fellows (n = 12) | 13x 1-hour sessions over one year | Pre-post feasibility study. Time of post-assessment is unclear but likely immediately post-intervention. | None | Education, positive role-modelling | Surveys assessed knowledge on racism in medicine; opinions, understanding, and comfort surrounding race and racism in medicine; as well as additional questions to solicit feedback on the curriculum itself | Assessed the feasibility of a year-long antiracism educational study. As it was mostly a feasibility study, postintervention surveying indicated a 15% increase in self-directed learning on related topics. However, interest in the curriculum actually decreased post-intervention by 14%, perhaps since participants now felt they had learned what they needed to. | present information from credible influence BCT BCIO:007075  inform about health consequences BCT BCIO:007063  increase awareness of behaviour BCT BCIO:007173  awareness of other peoples thoughts, feelings and actions BCT  BCIO:007072  instruct how to perform a behaviour BCT BCIO:007058  inform about antecedents BCT BCIO:007052 |
| Barrett et al. (2009)(31) | An inpatient unit, critical care unit, emergency department, and inpatient operating room  59 pre-intervention and 45 post-intervention nurses | Two 2-hour group sessions | Quasi-experimental pre-post design without control. Pre-intervention measures were 2-weeks prior to intervention and post-measures were 3-months after intervention. | None | Education and role playing, encouragement to become champions, teambuilding, seeking hospital accreditation | National Database of Nursing Quality Indicators (NDNQI) RN-RN interaction subscale, Group Cohesion Scale | The study assessed a team-building intervention to reduce lateral violence, using mixed methods. The intervention was found to improve group cohesion from 540 pts to 612 pts (p=0.037). | awareness of other peoples thoughts, feelings and actions BCT  BCIO:007072  prompt focus on self-identity BCT  BCIO:007157  provide feedback on behaviour BCT BCIO:007023  present information from credible influence BCT  BCIO:007075  deliver informational support BCT  BCIO:007042  prompt social comparison BCT BCIO:007073  identify self as role model BCT BCIO:007158  demonstrate the behaviour BCT  BCIO:007055  attend to discrepancy between current behaviour and goal BCT  BCIO:007012  prompt comparative imaging of future outcomes BCT BCIO:007070  instruct how to perform a behaviour BCT BCIO:007058 |
| Demarco, Roberts, and Chandler (2005)(37) | 5 graduate nursing student participants | 2 hours per week for 6 weeks | Pilot pre-post design without control. Interviews were as soon as possible post-intervention. | Oppressed group behaviour (Freire, 1990) | Group writing | Interview | Pilot study investigating a writing group’s ability to build group cohesion. The group writing drew on the ”Amherst Writers and Artists” method which did not focus on UB but nonetheless was intended to help cope with it. It explored participants’ responses qualitatively, finding it helped build a sense of social support, and recommended potential changes. No quantitative effectiveness testing. | provide feedback BCT BCIO:007022  awareness of other peoples thoughts, feelings and actions BCT BCIO:007072  advise sensory ways to change emotions BCT  BCIO:007167  provide feedback on outcome of behaviour BCT BCIO:007027  monitor emotional consequences BCT BCIO:007066  suggest how to perform behaviour BCT BCIO:007303  instruct how to perform a behaviour BCT  BCIO:007058  social support BCT  BCIO:007028 |
| Jenkins et al. (2011)(45) | 10 student leaders | 6-hour long sessions, monthly, for 6 months | Case study design | Unknown | Journal club / group writing | Weekly journal comments | Explores how 6 monthly hour-long journal club meetings may increase civility. Also incorporated journalling. Anecdotally, it found that participants became more aware and sought to become role models of civility. | identify self as role model BCT BCIO:007158  self-monitor outcome of behaviour BCT BCIO:007025  instruct how to perform a behaviour BCT BCIO:007058  deliver appraisal support BCT BCIO:007043  practise behaviour BCT  BCIO:007094 |
| Kang, Kim and Yun (2017)(47) | 40 hospital nurses | 20-hours over 10 sessions | Randomised controlled trial | 4 stages of cognitive rehearsal by Smith and non-violence communication (Rosenberg & Chopra, 2015) | Cognitive rehearsal, role playing, and communication training | Relationship Change Scale, Negative Acts Questionnaire-Revised, Brief Symptom Inventory-18, Yun’s nurse turn-  over intention tool | An RCT to investigate a cognitive rehearsal programme on workplace bullying. Post-intervention, there were significant differences in interpersonal relationships (F=6.21, p=.022) and turnover intention (F=5.55, p=.024) between groups, but not for workplace bullying. | context-specific repetition of alternative behaviour BCT  BCIO:007097  practise behaviour BCT  BCIO:007094  demonstrate the behaviour BCT  BCIO:007055  instruct how to perform a behaviour BCT BCIO:007058    awareness of other peoples thoughts, feelings and actions BCT  BCIO:007072  provide feedback BCT BCIO:007022  cue BCT  BCIO:007081 |
| Kousha et al. (2022)(49) | 80 emergency nurses | Five 2-hour sessions over 3 weeks | Single-blinded randomised controlled trial with two hospitals as groups (education-only vs. education and cognitive rehearsal). Post-collection of data was 1-month after training sessions. | Bandura’s social learning theory (1977) | Cognitive rehearsal, education, role-play | The Incivility Scale | The study, focused on incivility between emergency nurses, found that ANOVA and repeated measures ANOVA indicated overall incivility and supervisor incivility increased in the intervention group but decreased in the control group. There were no notable differences in instances of nurse-to-nurse incivility and physician incivility. | *Intervention:*  prompt mental rehearsal of successful performance BCT BCIO:007138  instruct how to perform a behaviour BCT  BCIO:007058  context-specific repetition of alternative behaviour BCT  BCIO:007097  practice behaviours  BCIO:007094  demonstrate the behaviour BCT  BCIO:007055  increase awareness of consequences BCT  BCIO:007062  increase awareness of behaviour BCT  BCIO:007173  present information from credible influence BCT BCIO:007075  *Control:*  deliver informational support BCT  BCIO:007042 |
| Lasater et al. (2015)(50) | 94 nursing staff | One 1-hour session comprising presentation and discussion on incivility, a 4-hour session on norm-setting and action planning, and a 2-hour simulation role playing session | Quasi-experimental study with no control group. Data collection occurred pre, post-session 1, post-session 2, post-session 3, >1 month post-session 3, 5 months after the last session, and 24-months after the last session. | Bandura’s social learning theory (1977) | Education, action planning, role playing | Nurse Incivility Scale (NIS)  New General Self-Effectiveness Scale (NGSE), Workplace Collective Effectiveness Scale (WCES),  National Database for Nursing Quality Indicators (NDNQI) | Study of a three-part educational intervention to see if it reduced incivility in two units of a hospital. It was effective in decreasing incidences of perceived incivility across all NIS subscales and self-effectiveness increased significantly. | increase awareness of behaviour BCT  BCIO:007173  deliver informational support BCT  BCIO:007042  inform about negative health consequences BCT BCIO:007179  increase salience of consequences BCT  BCIO:007068  inform about social consequences BCT  BCIO:007064  context-specific repetition of alternative behaviour BCT  BCIO:007097  demonstrate the behaviour BCT  BCIO:007055  practice behaviours  BCIO:007094  awareness of other peoples thoughts, feelings and actions BCT  BCIO:007072  substitute behaviour BCT BCIO:007095  identify self as role model BCT  BCIO:007158  agree on how to perform behaviour BCT BCIO:007051  cue BCT BCIO:007081  create behavioural contract BCT  BCIO:007014  prompt focus on self-identity BCT  BCIO:007157  provide feedback on behaviour BCT  BCIO:007023 |
| Nicotera, Magon and Wright (2014)(54) | 19 participants with 47 comparison sample | 6x 90 minute sessions | Pilot quasi-experimental pre-post design with comparison group. No randomisation. Post data collection was unclear. | Structurational divergence | Education, conflict management, role playing | Structurational divergence, role conflict, burnout, depression, bullying, beliefs about arguing, verbal aggressiveness, taking conflict personally (TCP, a set of attitudes predisposing one to internalize conflict), ambiguity intolerance, and conflict management styles (collaborative, avoidant, and aggressive), general conflict orientations, scales for direct constructive discussion and direct destructive discussion | Assesses a nursing conflict intervention using mixed pre and post-test methods with a control sample, which sought to reduce structurational divergence by raising awareness of it and teaching skills to manage it. They found that participants experienced better understanding of conflicts and how to sustain healthier relationships. The course significantly reduced destructive communication and improved constructive communication skills. | instruct how to perform a behaviour BCT BCIO:007058  prompt thinking related to successful performance BCT BCIO:007239  suggest different perspective on behaviour BCT  BCIO:007302  inform about antecedents BCT BCIO:007052  re-attribute cause BCT  BCIO:007053    restructure the social environment BCT  BCIO:050349  increase awareness of behaviour BCT  BCIO:007173  advise cognitive ways to increase positive emotions BCT  BCIO:050334  awareness of other peoples thoughts, feelings and actions BCT  BCIO:007072  demonstrate the behaviour BCT  BCIO:007055  practice behaviours  BCIO:007094 |
| O’Keeffe, Brennan and Doherty (2022)(57) | 203 participants in surgery | 1-day session with a 1-hour follow up e-learning course | Case study | Transformative learning theory conceptual framework | Role-playing, team-building, education, reflection | Online qualitative survey | Outlines an intervention based on a Transformative Learning Theory conceptual framework that draws upon role playing, simulations, case studies, reflection exercises, and peer-peer learning. Anecdotally, 85% of participants reported a perceived improvement in conflict management skills but effectiveness was not formally tested. | context-specific repetition of alternative behaviour BCT  BCIO:007097  practice behaviours  BCIO:007094  demonstrate the behaviour BCT  BCIO:007055  awareness of other peoples thoughts, feelings and actions BCT  BCIO:007072  increase awareness of behaviour BCT BCIO:007173  advise cognitive ways to reduce negative emotions BCT BCIO:050337  prompt social comparison BCT BCIO:007073  prompt thinking related to successful performance BCT  BCIO:007239  present information from credible influence BCT  BCIO:007075  deliver informational support BCT  BCIO:007042  provide feedback on behaviour BCT  BCIO:007023  tell to change behaviour BCT BCIO:007077 |
| Saxton (2012)(60) | 17 participants | Two-day programme | Pre-post study design without control group. Post-intervention data were collected immediately. Data collection also at 4-week follow-up. | Humanizing Nursing Communication Theory and Social Cognitive Theory | Communication training | Self-Effectiveness to Address Disruptive Behavior Scale | Development and evaluation of a communication skills intervention to improve perceived self-effectiveness of perioperative nurses using a pre-post design. Results suggested an improvement in self-effectiveness scores and participants reported the ability to address disruptive physician behaviour 71% of the time four weeks after the study intervention. | provide positive material consequence for outcome of behaviour BCT BCIO:007263  prompt focus on self-identity BCT  BCIO:007157  awareness of other peoples thoughts, feelings and actions BCT  BCIO:007072  context-specific repetition of alternative behaviour BCT  BCIO:007097  practice behaviours  BCIO:007094  demonstrate the behaviour BCT  BCIO:007055  increase awareness of behaviour BCT BCIO:007173 |
| ***Combined sessions plus extra*** | | | | | | | | |
| Chipps and McRury (2012)(33) | 16 staff members | 3 month | Quasi-experimental pre-post design without control with 4-month follow-up. Posttest given immediately post-intervention. | Einarsen’s (2000) theoretical framework on predisposing factors for workplace bullying | Education, peer learning, action planning, conflict management training, code of conduct, communication training | Negative Acts Questionnaire-Revised (NAQ-R). Log books given for daily documenting of bullying experiences. | This quasi-experimental study assessed pre-test and post-test an educational intervention to address workplace bullying. They noted an increase in bullying reports as a result (from <1 act weekly/daily to 1.6 acts weekly/daily), but it did help develop a learning community. | provide aversive consequence for behaviour BCT  BCIO:007241    increase salience of consequences BCT  BCIO:007068  increase awareness of consequences BCT  BCIO:007062    attend to discrepancy between current behaviour and goal BCT BCIO:007012  prompt comparative imaging of future outcomes BCT BCIO:007070  restructure the social environment BCT  BCIO:050349  set behaviour goal BCT BCIO:007003  adopt changed self-identity BCT BCIO:007160  goal strategising BCT BCIO:007008    arrange instrumental support BCT  BCIO:007035  arrange emotional support BCT BCIO:007036  deliver appraisal support BCT BCIO:007043  prompt focus on self-identity BCT BCIO:007157    provide feedback BCT BCIO:007022  instruct how to perform a behaviour BCT BCIO:007058  increase awareness of behaviour BCT BCIO:007173  inform about antecedents BCT BCIO:007052 |
| Dimarino (2011)(38) | Unknown | Unknown – ‘on demand’ sessions and code of conduct | Case study | None | Code of conduct, education | Reports anecdotally on organisational turnover and reports of violence. | Reporting of one ambulatory surgery centre’s approach to reducing lateral violence through education about lateral violence, and zero tolerance policies. Did not test effectiveness. | promise aversive consequence for behaviour BCT  BCIO:007189  deliver informational support BCT  BCIO:007042  increase awareness of consequences BCT  BCIO:007062  provide aversive material consequence for outcome of behaviour BCT BCIO:007245  advise to seek emotional support BCT BCIO:007031  advise to seek support BCT BCIO:007029  substitute behaviour BCT BCIO:007095  provide feedback BCT  BCIO:007022 |
| Kang and Jeong (2019)(46) | 72 hospital nurses | Two-hour familiarity session followed by 8-weeks on-demand usage (smartphone based) | Cluster quasi-randomised design with control group with pre, post (4-week post), and 8-week follow-up measurement | Non‐violent communication (Rosenberg & Chopra, 2015) | Cognitive rehearsal (smartphone based) | Negative Acts Questionnaire‐Revised, turnover intentions were measured using a modified version of “intent to quit” | Investigates a cognitive rehearsal smartphone-based intervention’s impact on bullying using a cluster quasi-randomised trial. It found that use of the app reduced nurses' person‐related (21.07+-8.38 to 15.41+-4.03 at 8 wks, ICU staff) and work‐related bullying experiences (11.19+-5.02 to 7.81+-3.20 at wks, ICU staff) and turnover intentions (3.52+-0.8 to 3.21+-0.71 at 8 wks, ICU staff) between groups, but did not reduce intimidation‐related bullying experiences. | Cue BCT BCIO:007081  advise behavioural ways to reduce negative emotions BCT  BCIO:050333  instruct how to perform a behaviour BCT BCIO:007058  substitute behaviour BCT BCIO:007095  arrange informational support BCT BCIO:007037  prompt mental rehearsal of successful performance BCT  BCIO:007138 |
| Parker et al. (2016)(59) | Unclear / organisation-wide | One away day & subsequent ongoing efforts of unclear overall duration | Case study design | Longo’s (2010) three-level intervention model: organization, leadership, and individual | Education, conflict management, leadership training, role modelling, cognitive rehearsal, code of conduct, championing / becoming agents of change, seeking Magnet status | Briles’ Sabotage Savvy Questionnaire | Explores how nurses at an acute care hospital were able to implement multiple interventions to reduce horizontal violence prevalence in the organisation. These included 13 total organisational, leadership, and individual level strategies. | provide aversive consequence for behaviour BCT  BCIO:007241  monitoring BCT BCIO:007017  instruct how to perform a behaviour BCT BCIO:007058  arrange support BCT BCIO:007034  arrange emotional support BCT BCIO:007036  present information from credible influence BCT  BCIO:007075  increase awareness of behaviour BCT BCIO:007173  increase awareness of consequences BCT BCIO:007062  awareness of other peoples thoughts, feelings and actions BCT BCIO:007072    action planning BCT BCIO:007010  goal strategising BCT BCIO:007008  substitute behaviour BCT BCIO:007095  prompt thinking related to successful performance BCT BCIO:007239  advise behavioural ways to increase positive emotions BCT BCIO:050350  advise behavioural ways to reduce negative emotions BCIO:050333  guide how to perform behaviour BCT BCIO:007050 |
| Stevens (2002)(64) | Unclear | Unclear | Case study design | None | Action planning, code of conduct, democratisation | Turnover rate | Informally explores the impact of a multi-strategy anti-bullying intervention in a large teaching hospital, delivered mostly through workshops for education, policies, supervisor training, and more. They anecdotally found a decrease in nursing turnover rate after 1 year but it could have been also a result of other factors and programmes. | provide aversive consequence for behaviour BCT  BCIO:007241  attend to discrepancy between current behaviour and goal BCT BCIO:007012  draw attention to incompatible beliefs BCT BCIO:007057  awareness of other peoples thoughts, feelings and actions BCT BCIO:007072  prompt focus on self-identity BCT BCIO:007157  instruct how to perform a behaviour BCT BCIO:007058  present information from credible influence BCT  BCIO:007075  provide feedback BCT  BCIO:007022  goal strategising BCT BCIO:007008  arrange support BCT BCIO:007034 |
| Thorsness and Sayers (1995)(65) | Approximately 100 surgical staff | Unclear | *Pre-post design without control group. Survey was conducted before and 2.5 years after intervention.* | Kilmann model for transforming organisations (1974) | Survey, code of conduct, action planning, communication skills training, workplace rearrangement, education | Kilmann-Saxton Culture-Gap Survey | Evaluation of a programme adopting a systems approach to cultivating a positive work environment for perioperative staff members. Intervention comprised making action plans for different staff groups, code of conduct, and cultural change efforts. Post-survey showed improvements in culture but statistical significance was not tested. | prompt thinking related to successful performance BCT  BCIO:007239  prompt comparative imaging of future outcomes BCT BCIO:007070    attend to discrepancy between current behaviour and goal BCT  BCIO:007012  draw attention to incompatible beliefs BCT BCIO:007057  awareness of other peoples thoughts, feelings and actions BCT BCIO:007072  provide aversive consequence for behaviour BCT  BCIO:007241  increase awareness of behaviour BCT  BCIO:007173  action planning BCT  BCIO:007010  goal strategising BCT  BCIO:007008  adopt positive self-identity BCT  BCIO:007161  restructure the social environment BCT  BCIO:050349  goal setting BCT  BCIO:007002  provide feedback on behaviour BCT  BCIO:007023  remove aversive social consequence for behaviour BCT BCIO:007290 |
| ***Professional accountability and reporting*** | | | | | | | |  |
| Baldwin et al. (2022)(29) | Three academic medical centres | Sept 2019 to Aug 2021 (2 years). | Descriptive study assessing feasibility of implementation. | None | Vanderbilt intervention (reporting and escalation system, informal and formal resolution, championing, peer messengers, seeking Magnet accreditation) | Statistics from message database. | Descriptive study analysing the types of reports received during the intervention to promote professionalism with nurses. 590 reports were received, of which most included more than one problematic behaviour. 76.5% of these messages were shared and completed. | instruct how to perform a behaviour BCT BCIO:007058  practice behaviours BCIO:007094  demonstrate the behaviour BCT  BCIO:007055  increase awareness of behaviour BCT  BCIO:007173  implore to change behaviour BCT  BCIO:007078  provide feedback on behaviour BCT BCIO:007023  guide how to perform behaviour BCT BCIO:007050  promise aversive consequence for behaviour  BCT BCIO:007189  provide aversive consequence for behaviour BCT BCIO:007241  awareness of other peoples thoughts, feelings and actions BCT  BCIO:007072  increase awareness of consequences BCT  BCIO:007062    inform about negative social consequences BCT  BCIO:007180  monitoring BCT BCIO:007017  substitute behaviour BCT BCIO:007095  prompt thinking related to successful performance BCT BCIO:007239 |
| Dixon-Woods et al. (2019)(39) | Organisation-wide at Johns Hopkins Medicine | Two-year period, 2014-2016 | ‘Case study’ approach | None | Reporting system and formal investigation | Interviews. | This study sought to improve employee’s ability to speak up about transgressive and disruptive behaviour at a John Hopkins Medicine hospital. It uses largely qualitative exploration of the results and highlights importance of leader commitment and intolerant culture. Makes suggestions for a testable approach to encouraging voice. Did not test effectiveness. | practise behaviour BCT  BCIO:007094  inform about negative emotional consequences BCT  BCIO:007177  promise aversive consequence for behaviour BCT BCIO:007189  provide aversive consequence for behaviour BCT  BCIO:007241  restructure the social environment BCT  BCIO:050349  remove aversive social consequence for behaviour BCT BCIO:007290  present information from credible influence BCT  BCIO:007075  identify self as role model BCT BCIO:007158  guide how to perform behaviour BCT BCIO:007050  deliver informational support BCT BCIO:007042  awareness of other peoples thoughts, feelings and actions BCT  BCIO:007072 |
| Ethos Combined (23,25,26,34) | Eight hospitals | 5 years | Mixed study designs. | Unknown | Ethos reporting system with peer messengers, informal resolution, formal investigation, training to enhance speaking up and role-modelling. | Studies report a range of outcome measures including understanding content of Ethos messages, experience of peer messengers, and reporting of UBs. | There was an overall reduction in the odds of staff experiencing incivility/bullying behaviours by 24%, and a 32% reduction in odds of experiencing extreme unprofessional behaviours in the previous 12 months. | set graded tasks BCT BCIO:007100  remove aversive social consequence for behaviour BCT BCIO:007290  provide positive consequence for behaviour BCT BCIO:007252  provide feedback on behaviour BCT  BCIO:007023  deliver support BCT BCIO:007039  awareness of other peoples thoughts, feelings and actions BCT  BCIO:007072  increase awareness of consequences BCT  BCIO:007062  action planning BCT  BCIO:007010  provide aversive consequence for behaviour BCT BCIO:007241    promise aversive consequence for behaviour BCT  BCIO:007189  prompt thinking related to successful performance BCT BCIO:007239  instruct how to perform a behaviour BCT BCIO:007058  monitoring BCT BCIO:007017  arrange support BCT BCIO:007034  identify self as role model BCT BCIO:007158  increase awareness of behaviour BCT  BCIO:007173  promise positive material consequence for behaviour BCT BCIO:007209  advise to seek support BCT BCIO:007029  arrange emotional support BCT BCIO:007036  increase salience of consequences BCT BCIO:007068  consider pros and cons BCT BCIO:007069  increase awareness of others approval BCT BCIO:007074  social support BCT  BCIO:007028  deliver informational support BCT BCIO:007042  induce anticipated regret BCT  BCIO:007067    demonstrate the behaviour BCT  BCIO:007055  provide feedback BCT BCIO:007022 |
| Hickson et al. (2007)(44) | Unknown | Variable, depends on requirements | Case study design | Unknown | Vanderbilt approach for graduated intervention (reporting system, informal conversation, awareness, leader-led action planning, formal investigation) & other variable strategies as required (e.g. communication training, leadership programmes, use of messengers) | Reports statistics on reporting system use and types of complaint. | Outlines and reviews the Vanderbilt approach to identifying, measuring, and addressing UB using four graduated interventions. Did not assess effectiveness. | consider pros and cons BCT BCIO:007069  instruct how to perform a behaviour BCT BCIO:007058  set behaviour goal BCT  BCIO:007003  provide feedback on behaviour BCT BCIO:007023  promise aversive consequence for behaviour BCT BCIO:007189  awareness of other peoples thoughts, feelings and actions BCT BCIO:007072  prompt focus on self-identity BCT BCIO:007157  identify self as role model BCT BCIO:007158  increase awareness of behaviour BCT BCIO:007173  provide positive consequence for behaviour BCT BCIO:007252  promise aversive material consequence for outcome of behaviour BCIO:007245  monitoring BCT BCIO:007017  promise aversive material consequence for behaviour BCT BCIO:007192  practice behaviours BCIO:007094  demonstrate the behaviour BCT  BCIO:007055  prompt thinking related to successful performance BCT BCIO:007239  guide how to perform behaviour BCT BCIO:007050 |
| McKenzie et al. (2019)(53) | 21 healthcare staff pre-to-post | 18 months into a 3-year intervention | ‘Case study’ design | Unknown | Vanderbilt approach (education, reporting system, champions, action plans, graduated informal to formal resolution) | Interviews and Safety Attitudes Questionnaire (SAQ) | Investigated factors affecting implementation of a multistrategy intervention using education, reporting systems with graduated intervention processes, safety champions, and action plans, to tackle unprofessional behaviour. They found that leader role modelling, work condition modification, confidence in accountability systems, and responsiveness enhanced the intervention implementation. | increase awareness of behaviour BCT  BCIO:007173  increase awareness of consequences BCT  BCIO:007062    monitoring BCT BCIO:007017  promise aversive consequence for behaviour BCT BCIO:007189  provide aversive consequence for outcome of behaviour BCT  BCIO:007246  promise positive consequence for alternative behaviour BCT BCIO:007204  remove aversive social consequence for behaviour BCT BCIO:007290  identify self as role model BCT BCIO:007158  action planning BCT  BCIO:007010 |
| Speck et al. (2014)(61) | Three teaching hospitals | 4+ years | Case study design | Unknown | Variation on the Vanderbilt reporting system with graduated escalation from informal resolution (peer, then manager) to formal investigation, championing | Reports on professionalism committee statistics and vignettes | Assessment of a professionalism committee approach to tackling unprofessional behaviour across three large teaching hospitals. In this variation, department chairs were able to report individuals to the committee rather than any staff member. It found that it was able to identify early specific behavioural issues and refer them appropriately. | monitoring BCT BCIO:007017  deliver support BCT BCIO:007039  awareness of other peoples thoughts, feelings and actions BCT  BCIO:007072  action planning BCT  BCIO:007010  promise aversive consequence for behaviour BCT BCIO:007189  adopt changed self-identity BCT  BCIO:007160  arrange support BCT BCIO:007034  provide aversive consequence for behaviour BCT  BCIO:007241 |
| Webb et al. (2016)(67) | Three hospitals | 2 years for study data (but programme running for 9 years) | Case study/ evaluation | Unknown | Vanderbilt reporting system with graduated escalation from informal resolution (peer, then manager) to formal investigation, championing | Reports on statistics from the reporting system such as number of reports and escalations | Presents a feasibility study of the Co-Worker Observation Reporting System implemented by Vanderbilt University Medical Centre to reduce disrespectful and unsafe behaviours. They found that it was feasible, requiring organisation-wide implementation and found that most workers self-regulate after being informed a report was received about them. | monitoring BCT BCIO:007017  identify self as role model BCT BCIO:007158  awareness of other peoples thoughts, feelings and actions BCT  BCIO:007072  increase awareness of consequences BCT  BCIO:007062  consider pros and cons BCT  BCIO:007069  attend to discrepancy between current behaviour and goal BCT  BCIO:007012  prompt focus on self-identity BCT  BCIO:007157  remove aversive social consequence for behaviour BCT BCIO:007290    provide feedback on behaviour BCT BCIO:007023  prompt thinking related to successful performance BCT BCIO:007239  advise to seek support BCT BCIO:007029  inform about health consequences BCT BCIO:007063  promise aversive consequence for behaviour BCT BCIO:007189 |
| ***Structured culture change*** | | | | | | | | |
| Armstrong (2017)(27) | 9 nurses | Two 8-hour sessions to train facilitators  Four weeks total with one meeting per week. Sessions lasted 20-30 mins. | Pre-post design without control. Assessment two-weeks post-intervention. | Model for Improvement by Langley (1996) | CREW (Education, teambuilding exercises, roleplaying) | Workplace Incivility Scale, Confidence Scale | Quantitative investigation of an intervention in a rural hospital to reduce nursing workplace incivility. Findings indicated no change in experience of incivility but a greater ability to respond to incivility (there was a statistically significant improvement in the posttest mean score (M = 85.56, SD =20.07, t (8) = -4.667, p =.002), when compared to the pretest mean score (M = 62.22, SD = 18.56). | suggest different perspective on behaviour BCT  BCIO:007302  increase awareness of behaviour BCT BCIO:007173  guide how to perform behaviour BCT BCIO:007050  facilitate alternative goal-directed activity BCT BCIO:007171  awareness of other peoples thoughts, feelings and actions BCT  BCIO:007072  attend to discrepancy between current behaviour and goal BCT  BCIO:007012  instruct how to perform a behaviour BCT BCIO:007058  deliver informational support BCT  BCIO:007042  practice behaviours BCIO:007094  demonstrate the behaviour BCT  BCIO:007055    present information from credible influence BCT  BCIO:007075 |
| Laschinger et al. (2012)(51) | 8 units with 33 controls | 6 months, selecting strategies from the CREW toolkit as appropriate | Quasi-experimental design using randomised units. Pre and post assessment. Post-assessment timing is unclear. | Kanter’s (1977, 1993) Theory of Structural Power in Organizations | CREW intervention (espousing values by leadership / role modelling, education, signing code of conduct pledge, surveying civility, action planning, role playing, training on assertiveness, communication, conflict resolution). | Structural empowerment was measured using four subscales of the Conditions for Work Effectiveness Questionnaire-II, five items from the Workplace Incivility Scale, Six items from Cook and Wall’s (1980) Interpersonal Trust at Work Scale | Investigates the impact of the CREW intervention over 6 months with 8 intervention units and 33 control units. A significant interaction of time by intervention was found for the access to support and resources empowerment structures, total empowerment, supervisor incivility, and trust in management | prompt social comparison BCT  BCIO:007073  arrange support BCT BCIO:007034  instruct how to perform a behaviour BCT BCIO:007058  awareness of other peoples thoughts, feelings and actions BCT  BCIO:007072  action planning BCT  BCIO:007010  set behaviour goal BCT BCIO:007003    goal strategising BCT BCIO:007008 |
| Leiter et al. (2011)(52) | 1,173 workers across 41 units | 6-months | Quasi-experimental design using randomised units. Pre and post assessment. Post-assessment timing is unclear. | Social interactions at work | CREW intervention (espousing values by leadership / role modelling, education, signing code of conduct pledge, surveying civility, action planning, role playing, training on assertiveness, communication, conflict resolution, leadership). | CREW Civility Scale, The 10-  item Workplace Incivility Scale and  an additional dimension of instigated workplace incivility was included Blau and Andersson  (2005), respect was measured using two items from the  Esteem Reward section of the Effort-Reward Imbalance Questionnaire, trust in management was measured  by six items from Cook and Wall’s (1980) Interpersonal Trust at Work Scale, Emotional Exhaustion and Cynicism subscales  of the Maslach Burnout Inventory-General Survey, three items were modified from the  Turnover Intentions measure developed by Kelloway, Gottlieb, and Barham (1999), Professional Effectiveness was measured using the Professional Effectiveness scale of the MBI-GS, Two items from the Affective Commitment Scale (Allen & Meyer, 1990), job satisfaction (Hackman & Oldham, 1975; Tsui, Egan & O’Reilly, 1992), absenteeism from institutional data. | Assesses the effect of 6 months of the CREW intervention and found that greater group x time interactions were found in the intervention group for civility, supervisor incivility, respect, cynicism, job satisfaction, and management trust, and absences. | provide feedback BCT BCIO:007022  arrange support BCT BCIO:007034  action planning BCT  BCIO:007010  set behaviour goal BCT BCIO:007003  goal strategising BCT BCIO:007008  identify self as role model BCT BCIO:007158  present information from credible influence BCT  BCIO:007075  create behavioural contract BCT  BCIO:007014  increase awareness of behaviour BCT  BCIO:007173  deliver informational support BCT  BCIO:007042  restructure the social environment BCT  BCIO:050349  deliver support BCT BCIO:007039  prompt social comparison BCT  BCIO:007073  prompt thinking related to successful performance BCT BCIO:007239  awareness of other peoples thoughts, feelings and actions BCT  BCIO:007072  provide positive consequence for behaviour BCT  BCIO:007252  promise positive material consequence for behaviour BCT BCIO:007209  instruct how to perform a behaviour BCT BCIO:007058  practice behaviours BCIO:007094  guide how to perform behaviour BCT BCIO:007050  demonstrate the behaviour BCT  BCIO:007055  record behaviour without feedback BCT  BCIO:007019  monitoring BCT BCIO:007017 |
| Osatuke et al. (2009)(58) | 647 post-intervention CREW participants and 680 comparison (total 34 workgroups) | Flexible/various | Quasi-experimental pre-post design with control group but no randomisation. The Civility scale for CREW was  administered at CREW-1 sites in September 2005 and July 2006, and at CREW-2 sites in February 2006 and March 2007. Comparison site data were retrospectively matched to intervention sites for respective years. | National Center for Organization Development (NCOD) practice model. Prototype approach. | CREW intervention (survey, action planning, various training e.g., on communication, education) | CREW civility scale | Preliminary evaluation of a nationwide Veterans Health Administration interventions called CREW across 23 sites. It found significant pre to post-intervention changes in civility compared to none at comparison sites. | action planning BCT  BCIO:007010  set behaviour goal BCT BCIO:007003  goal strategising BCT BCIO:007008  increase awareness of behaviour BCT  BCIO:007173  present information from credible influence BCT  BCIO:007075  suggest different perspective on behaviour BCT  BCIO:007302  awareness of other peoples thoughts, feelings and actions BCT  BCIO:007072  monitoring BCT  BCIO:007017  provide feedback BCT BCIO:007022  deliver support BCT BCIO:007039  arrange support BCT BCIO:007034  arrange informational support BCT  BCIO:007037  advise to keep outcome goal in mind BCT BCIO:007142  deliver instrumental support BCT BCIO:007040  guide how to perform behaviour BCT BCIO:007050 |
