# Supplementary material for: Systematically analyzing behavior change techniques used in 44 interventions to reduce unprofessional behavior between healthcare staff
Source: Transl Behav Med. 2025 Oct 15;15(1):ibaf058. doi: 10.1093/tbm/ibaf058 (PMC12527449; doi:10.1093/tbm/ibaf058)
Supplement: ibaf058_Supplementary_Data [file ibaf058_supplementary_data.zip › Supplementary File 3 - BCT proportion graph.docx]

Figure 1. Proportion of BCTs used within each intervention type (e.g. 15.4% of BCTs used by single session interventions were “guide how to perform behaviour BCTs”).
