# Supplementary material for: Systematically analyzing behavior change techniques used in 44 interventions to reduce unprofessional behavior between healthcare staff
Source: Transl Behav Med. 2025 Oct 15;15(1):ibaf058. doi: 10.1093/tbm/ibaf058 (PMC12527449; doi:10.1093/tbm/ibaf058)
Supplement: ibaf058_Supplementary_Data [file ibaf058_supplementary_data.zip › Supplementary File 1 - Search strategy.docx]

**Google Scholar (Via Harzing’s Publish or Perish)**

Top 20 entries included.

Search 1

bullying | harassment | discrimination|unprofessional AND workplace|worker|staff AND model|framework|concept|idea|opinion|theory|view|perception|attitude|theories [Searched in Publish or Perish Title Field]

Search 2

interventions|strategies|techniques|program|programs|programme|programmes AND unprofessional|bullying|harassment|discrimination AND nurse|doctor|paramedic|hospital|ambulance|staff|professional AND emergency|acute|trauma [searched in Publish or Perish Keywords field]

**CINAHL (EBSCOhost)**

Date searched: 25-08-2022

Records found:

# Query Results

S39 S37 AND S38

S38 (MH "United States+") OR AF ( USA or "united states" or america* ) OR TI ( USA or "united states" or america* ) OR AB ( USA or "united states" or america* )

S37 S35 NOT S36

S36 TX ( ("elder mistreat*" or "elder abuse*" or "elder neglect*") )

S35 S33 NOT S34

S34 ( ( (MH "Child") OR (MH "Adolescence+") OR (MH "Minors (Legal)") ) NOT (MH "Adult+") )

S33 S22 NOT S32

S32 S28 NOT S31

S31 S29 OR S30

S30 (MH "Australia+") OR (MH "Europe") OR (MH "Austria") OR (MH "Baltic States+") OR (MH "Belgium") OR (MH "Canada+") OR (MH "Chile") OR (MH "Colombia") OR (MH "Costa Rica") OR (MH "Czech Republic") OR (MH "Scandinavia+") OR (MH "France") OR (MH "Germany+") OR (MH "Greece") OR (MH "Hungary") OR (MH "Iceland") OR (MH "Ireland") OR (MH "Italy") OR (MH "Israel") OR (MH "Japan") OR (MH "South Korea") OR (MH "Luxembourg") OR (MH "Mexico") OR (MH "Netherlands") OR (MH "New Zealand") OR (MH "North America") OR (MH "Poland") OR (MH "Portugal") OR (MH "Slovakia") OR (MH "Slovenia") OR (MH "Spain") OR (MH "Switzerland") OR (MH "Turkey") OR (MH "United Kingdom+") OR (MH "United States+")

S29 (MH "Developed Countries") or (MH "European Union") or (MH "Organisation for Economic Co-Operation and Development")

S28 (S23 OR S24 OR S25 OR S26 OR S27)

S27 (MH "Africa+")

S26 (MH "Asia, Southeastern+") OR (MH "China+") OR (MH "Hong Kong") OR (MH "Macao") OR (MH "Mongolia") OR (MH "North Korea") OR (MH "Taiwan") OR (MH "Atlantic Islands") OR (MH "Indian Ocean Islands+") OR (MH "Melanesia+") OR (MH "Micronesia+") OR (MH "Polynesia+")

S25 (MH "Bangladesh") OR (MH "Bhutan") OR (MH "India") OR (MH "Yemen") OR (MH "United Arab Emirates") OR (MH "Syria") OR (MH "Saudi Arabia") OR (MH "Qatar") OR (MH "Oman") OR (MH "Lebanon") OR (MH "Kuwait") OR (MH "Jordan") OR (MH "Iraq") OR (MH "Iran") OR (MH "Bahrain") OR (MH "Afghanistan") OR (MH "Nepal") OR (MH "Pakistan") OR (MH "Sri Lanka") OR (MH "Asia, Central+")

S24 (MH "Argentina") OR (MH "Bolivia") OR (MH "Brazil") OR (MH "Ecuador") OR (MH "French Guiana") OR (MH "Guyana") OR (MH "Paraguay") OR (MH "Peru") OR (MH "Suriname") OR (MH "Uruguay") OR (MH "Venezuela") OR (MH "Belize") OR (MH "El Salvador") OR (MH "Guatemala") OR (MH "Honduras") OR (MH "Nicaragua") OR (MH "Panama+") or (MH "West Indies+")

S23 (MH "Albania") OR (MH "Andorra") OR (MH "Armenia") OR (MH "Azerbaijan") or (MH "Byelarus") OR (MH "Bosnia-Herzegovina") OR (MH "Croatia") OR (MH "Bulgaria") OR (MH "Georgia (Republic)") OR (MH "Gibraltar") OR (MH "Liechtenstein") OR (MH "Macedonia (Republic)") OR (MH "Moldova") OR (MH "Monaco") OR (MH "Romania") OR (MH "Russia") OR (MH "San Marino") OR (MH "Serbia") OR (MH "Ukraine") OR (MH "Yugoslavia")

S22 S5 AND S21

S21 S16 OR S17 OR S18 OR S19 OR S20

S20 TI ( (staff or employee* or work* or nurs* or doctor#) n5 (sexism or sexist or ableis* or racism or racist or (racial n3 abus*)) ) OR AB ( (staff or employee* or work* or nurs* or doctor#) n5 (sexism or sexist or ableis* or racism or racist or (racial n3 abus*)) ) OR SU ( (staff or employee* or work* or nurs* or doctor#) n5 (sexism or sexist or ableis* or racism or racist or (racial n3 abus*)) )

S19 TI ( ((staff or employee* or work* or nurs* or doctor#) n6 ((gender or disabilit* or disabled) n4 (inequalit* or equalit*))) OR ((staff or employee* or nurs* or doctor?) n1 (aggressi* or abus*))) OR AB ( ((staff or employee* or work* or nurs* or doctor#) n6 ((gender or disabilit* or disabled) n4 (inequalit* or equalit*))) OR ((staff or employee* or nurs* or doctor?) n1 (aggressi* or abus*)) ) OR SU ( ((staff or employee* or work* or nurs* or doctor#) n6 ((gender or disabilit* or disabled) n4 (inequalit* or equalit*))) OR ((staff or employee* or nurs* or doctor?) n1 (aggressi* or abus*)) )

S18 ((MH "Workplace Violence") or (MH "Aggression") or (MH "Prejudice+") or (MH "Discrimination+") or (MH "Dehumanization") OR (MH "Oppressed Group Behavior") OR (MH "Coercion")) AND ( (MH "Interprofessional Relations+") or (MH "Intraprofessional Relations"))

S17 TI ( ((staff or employee* or work* or nurs* or doctor#) n4 (hostil* or undermin* or discrimination or discriminatory or discriminated or humiliat*) ) OR ((staff or employee* or work* or nurs* or doctor#) n4 (WPV or violen* or victim* or coerci*) n3 among*) OR "abusive supervision" OR (workplace n2 (conflict* or aggressi* or abus*)) ) OR AB ( ((staff or employee* or work* or nurs* or doctor#) n4 (hostil* or undermin* or discrimination or discriminatory or discriminated or humiliat*) ) OR ((staff or employee* or work* or nurs* or doctor#) n4 (WPV or violen* or victim* or coerci*) n3 among*) OR "abusive supervision" OR (workplace n2 (conflict* or aggressi* or abus*)) ) OR SU ( ((staff or employee* or work* or nurs* or doctor#) n4 (hostil* or undermin* or discrimination or discriminatory or discriminated or humiliat*) ) OR ((staff or employee* or work* or nurs* or doctor#) n4 (WPV or violen* or victim* or coerci*) n3 among*) OR "abusive supervision" OR (workplace n2 (conflict* or aggressi* or abus*)) )

S16 S9 AND S15

S15 S10 OR S11 OR S12 OR S13 OR S14

S14 (MH "Health Personnel+") or (MH "Students, Health Occupations+") or (MH "Internship and Residency") OR (MH "Education, Graduate") OR (MH "Teamwork")

S13 TI ( (student# n2 (health* or clinic* or medic*)) OR employee# or worker# or Staff or personnel or practitioner# or professional# or workforce* or workplace* or "work place*" or worksite* or "work site*" or "work setting*" OR team*) OR AB ( (student# n2 (health* or clinic* or medic*)) OR employee# or worker# or Staff or personnel or practitioner# or professional# or workforce* or workplace* or "work place*" or worksite* or "work site*" or "work setting*" OR team*) OR SU ( (student# n2 (health* or clinic* or medic*)) OR employee# or worker# or Staff or personnel or practitioner# or professional# or workforce* or workplace* or "work place*" or worksite* or "work site*" or "work setting*" OR team*)

S12 TI ( Pathologist# or P#ediatrician# or Physiatrist# or Psychiatrist# or Pulmonologist# or Radiographer# or Radiologist# OR assistant# or cleaner# or ancillary or porter# or auxillary or auxillaries or administrator# or secretary or secretaries or receptionist# or technician# ) OR AB ( Pathologist# or P#ediatrician# or Physiatrist# or Psychiatrist# or Pulmonologist# or Radiographer# or Radiologist# OR assistant# or cleaner# or ancillary or porter# or auxillary or auxillaries or administrator# or secretary or secretaries or receptionist# or technician# ) OR SU ( Pathologist# or P#ediatrician# or Physiatrist# or Psychiatrist# or Pulmonologist# or Radiographer# or Radiologist# OR assistant# or cleaner# or ancillary or porter# or auxillary or auxillaries or administrator# or secretary or secretaries or receptionist# or technician# )

S11 TI ( Audiologist# or Anatomist# or Allergist# or An#esthetist# or An#esthesiologist# or Cardiologist# or Dieti#ian# or Endocrinologist# or Gastroenterologist# or GP# or Geriatrician# or Hospitalist# or Oncologist# OR Ophthalmologist# or Otolaryngologist# ) OR AB ( Audiologist# or Anatomist# or Allergist# or An#esthetist# or An#esthesiologist# or Cardiologist# or Dieti#ian# or Endocrinologist# or Gastroenterologist# or GP# or Geriatrician# or Hospitalist# or Oncologist# OR Ophthalmologist# or Otolaryngologist# ) OR SU ( Audiologist# or Anatomist# or Allergist# or An#esthetist# or An#esthesiologist# or Cardiologist# or Dieti#ian# or Endocrinologist# or Gastroenterologist# or GP# or Geriatrician# or Hospitalist# or Oncologist# OR Ophthalmologist# or Otolaryngologist# )

S10 TI ( ( nurs* or midwif* or midwiv or paramedic# or doctor# or physician# or clinician# or surgeon# or consultant# OR medic# or intern# or resident# or Therapist# or Pharmacist# or Optometrist# or Nutritionist# or Dentist# or Physiotherapist# ) ) OR AB ( ( nurs* or midwif* or midwiv or paramedic# or doctor# or physician# or clinician# or surgeon# or consultant# OR medic# or intern# or resident# or Therapist# or Pharmacist# or Optometrist# or Nutritionist# or Dentist# or Physiotherapist# ) ) OR SU ( ( nurs* or midwif* or midwiv or paramedic# or doctor# or physician# or clinician# or surgeon# or consultant# OR medic# or intern# or resident# or Therapist# or Pharmacist# or Optometrist# or Nutritionist# or Dentist# or Physiotherapist# ) )

S9 S6 OR S7 OR S8

S8 TI ( ( ((lateral* or horizontal*) n2 violence) ) OR ( ((transgressive or disruptive or unprofessional) n2 behavio#r*) ) OR (professional* n2 misconduct) OR ( "negative behavio*" or "negative act" ) ) OR AB ( ( ((lateral* or horizontal*) n2 violence) ) OR ( ((transgressive or disruptive or unprofessional) n2 behavio#r*) ) OR (professional* n2 misconduct) OR ( "negative behavio*" or "negative act" ) ) OR SU ( ( ((lateral* or horizontal*) n2 violence) ) OR ( ((transgressive or disruptive or unprofessional) n2 behavio#r*) ) OR (professional* n2 misconduct) OR ( "negative behavio*" or "negative act" ) )

S7 TI ( bully* or harass* or intimidat* or micro-aggress* or microaggress* or incivil* or uncivil* or rude* or mistreat* or mobbing or hazing or gaslight* or malic* or (Hidden n5 (aggressi* or abus* or violenc*))) OR AB ( bully* or harass* or intimidat* or micro-aggress* or microaggress* or incivil* or uncivil* or rude* or mistreat* or mobbing or hazing or gaslight* or malic* or (Hidden n5 (aggressi* or abus* or violenc*))) OR SU ( bully* or harass* or intimidat* or micro-aggress* or microaggress* or incivil* or uncivil* or rude* or mistreat* or mobbing or hazing or gaslight* or malic* or (Hidden n5 (aggressi* or abus* or violenc*)))

S6 (MH "Disruptive Behavior") or (MH "Bullying+") OR (MH "Emotional Abuse") OR (MH "Verbal Abuse") or (MH "Sexual Harassment") or (MH "Professional Misconduct") or (MH "Incivility") or (MH "Scapegoating")

S5 S1 OR S2 OR S3 OR S4 520,545

S4 TI ( ( (critical* n2 (care or ill*)) ) OR ( (urgent n2 (care or service* or medic*)) ) OR "intensive care" OR paramedic*) OR AB ( ( (critical* n2 (care or ill*)) ) OR ( (urgent n2 (care or service* or medic*)) ) OR "intensive care" OR paramedic*) OR SU ( ( (critical* n2 (care or ill*)) ) OR ( (urgent n2 (care or service* or medic*)) ) OR "intensive care" OR paramedic*)

S3 TI ( ( ((trauma* or ambulan*) n4 (care or service* or ill* or unit* or centre* or centre* or department* or setting)) ) ) OR AB ( ( ((trauma* or ambulan*) n4 (care or service* or ill* or unit* or centre* or centre* or department* or setting)) ) ) OR SU ( ( ((trauma* or ambulan*) n4 (care or service* or ill* or unit* or centre* or centre* or department* or setting)) ) )

S2 TI ( ( ((emergenc* or acute*) n4 (care or service* or health* or ill* or treat* or medic* or unit* or centre* or centre* or department* or setting* or ward#)) ) ) OR AB ( ( ((emergenc* or acute*) n4 (care or service* or health* or ill* or treat* or medic* or unit* or centre* or centre* or department* or setting* or ward#)) ) ) OR SU ( ( ((emergenc* or acute*) n4 (care or service* or health* or ill* or treat* or medic* or unit* or centre* or centre* or department* or setting* or ward#)) ) )

S1 (MH "Emergency Medical Services+") or (MH "Emergency Treatment+") or (MH "Emergency Care") or (MH "Airway Management+") or (MH "Ambulatory Care") OR (MH "Acute Care") or (MH "Critical Care+") OR (MH "Perioperative Care") OR (MH "Preoperative Care+") or (MH "Critical Care Nursing+") or (MH "Emergency Nursing+")

**EMBASE**

1 emergency health service/ or emergency medical dispatch/ or hospital emergency service/ or psychiatric emergency service/

2 emergency treatment/ or evidence based emergency medicine/

3 emergency care/ or advanced trauma life support/ or emergency ward/

4 respiration control/ or exp assisted ventilation/ or exp artificial ventilation/

5 exp ambulatory care/ or exp intensive care/

6 perioperative nursing/ or exp perioperative period/ or exp preoperative care/

7 exp hotline/ or poison center/ or exp ambulance/

8 exp intensive care nursing/ or emergency nursing/

9 (emergenc* adj5 (care or service* or health* or ill* or treat* or medic* or unit* or centre* or centre* or department* or setting*)).tw,kf.

10 (acute* adj5 (care or service* or health* or ill* or treat* or medic* or unit* or centre* or centre* or department* or setting* or ward?)).tw,kf.

11 (trauma* adj5 (care or service* or ill* or unit* or centre* or centre* or department*)).tw,kf.

12 (ambula* adj5 (care or service* or unit* or centre* or centre* or department* or setting*)).tw,kf.

13 (critical* adj2 (care or ill*)).tw,kf.

14 (urgent adj3 (care or service* or medic*)).tw,kf.

15 "intensive care".tw,kf.

16 paramedic*.tw,kf.

17 or/1-16 [Acute Care or Ambulance services]

18 agonistic behavior/

19 exp bullying/

20 disruptive behavior/

21 harassment/ or non-sexual harassment/ or exp online harassment/ or exp sexual harassment/

22 incivility/

23 microaggression/

24 professional misconduct/

25 exp hostility/

26 bully*.tw,kf.

27 harass*.tw,kf.

28 intimidat*.tw,kf.

29 (lateral* adj2 violence).tw,kf.

30 (horizontal* adj2 violence).tw,kf.

31 (transgressive adj3 behavio?r*).tw,kf.

32 (disruptive adj3 behavio?r*).tw,kf.

33 (unprofessional adj3 behavio?r*).tw,kf.

34 (micro-aggress* or microaggress*).tw,kf.

35 incivil*.tw,kf.

36 uncivil*.tw,kf.

37 rude*.tw,kf.

38 mistreat*.tw,kf.

39 (professional* adj3 misconduct).tw,kf.

40 mobbing.tw,kf.

41 (negative behavio* or negative act?).tw,kf.

42 hazing.tw,kf.

43 (gaslight* or malic*).tw,kf.

44 (Hidden adj5 (aggressi* or abus* or violenc*)).tw,kf.

45 or/18-44 [Unprofessional behaviours]

46 exp health care personnel/

47 exp health student/

48 exp medical education/

49 public relations/

50 workplace/

51 teamwork/

52 (nurs* or midwif* or midwiv*).tw,kf.

53 paramedic?.tw,kf.

54 (doctor? or physician? or clinician? or surgeon? or consultant?).tw,kf.

55 (student? adj2 (medic* or health* or clinic*)).tw,kf.

56 intern?.tw,kf.

57 resident?.tw,kf.

58 (Therapist? or Pharmacist? or Optometrist? or Nutritionist? or Dentist? or Physiotherapist?).tw,kf.

59 (Audiologist? or Anatomist? or Allergist? or An?esthetist? or An?esthesiologist? or Cardiologist? or Dieti#ian? or Endocrinologist? or Gastroenterologist? or GP? or Geriatrician? or Hospitalist? or Oncologist?).tw,kf.

60 (Ophthalmologist? or Otolaryngologist? or Pathologist? or P?ediatrician? or Physiatrist? or Psychiatrist? or Pulmonologist? or Radiographer? or Radiologist?).tw,kf.

61 medic?.tw,kf.

62 assistant?.tw,kf.

63 (cleaner? or ancillary or porter?).tw,kf.

64 (auxillary or auxillaries or administrator? or secretary or secretaries or receptionist? or technician?).tw,kf.

65 (employee? or worker? or staff or personnel or practitioner? or professional? or workforce* or team*).tw,kf. /freq=2

66 (workplace* or "work place*" or worksite* or "work site*" or "work setting*").tw,kf.

67 or/46-66 [Staff]

68 45 and 67 [UB and Staff search 1]

69 public relations/ and (aggression/ or aggressiveness/ or prejudice/ or exp social discrimination/ or coercion/)

70 ((staff or employee* or work* or nurs* or doctor?) adj8 (WPV or violen*) adj5 among*).tw,kf.

71 ((staff or employee* or work* or nurs* or doctor?) adj8 Victim* adj5 among*).tw,kf.

72 ((staff or employee* or work* or nurs* or doctor?) adj8 (humiliat* or hostil*)).tw,kf.

73 ((staff or employee* or work* or nurs* or doctor?) adj3 undermin*).tw,kf.

74 ((staff or employee* or work* or nurs* or doctor?) adj6 (discrimination or discriminatory or discriminated)).tw,kf.

75 "abusive supervision".tw,kf.

76 (workplace adj3 (conflict* or aggressi* or abus*)).tw,kf.

77 ((staff or employee* or work* or nurs* or doctor?) adj5 (gender adj2 (inequalit* or equalit*))).tw,kf.

78 ((staff or employee* or work* or nurs* or doctor?) adj5 (racism or racist or (racial adj3 abus*))).tw,kf.

79 ((staff or employee* or work* or nurs* or doctor?) adj5 (sexism or sexist)).tw,kf.

80 ((staff or employee* or work* or nurs* or doctor?) adj8 ((disabilit* or disabled) adj5 (inequalit* or equalit*))).tw,kf.

81 ((staff or employee* or work* or nurs* or doctor?) adj8 ableis*).tw,kf.

82 ((staff or employee* or work* or nurs* or doctor?) adj8 coerci* adj5 among*).tw,kf.

83 ((staff or employee* or nurs* or doctor?) adj1 (aggressi* or abus*)).tw,kf.

84 or/69-83 [UB and staff search 2]

85 68 or 84 [UB among Staff final search]

86 17 and 85 [UB among Staff in Acute Care]

87 afghanistan/ or africa/ or "africa south of the sahara"/ or albania/ or algeria/ or andorra/ or angola/ or argentina/ or "antigua and barbuda"/ or armenia/ or exp azerbaijan/ or bahamas/ or bahrain/ or bangladesh/ or barbados/ or belarus/ or belize/ or benin/ or bhutan/ or bolivia/ or borneo/ or exp "bosnia and herzegovina"/ or botswana/ or exp brazil/ or brunei darussalam/ or bulgaria/ or burkina faso/ or burundi/ or cambodia/ or cameroon/ or cape verde/ or central africa/ or central african republic/ or chad/ or exp china/ or comoros/ or congo/ or cook islands/ or coted'ivoire/ or croatia/ or cuba/ or cyprus/ or democratic republic congo/ or djibouti/ or dominica/ or dominican republic/ or ecuador/ or el salvador/ or egypt/ or equatorial guinea/ or eritrea/ or eswatini/ or ethiopia/ or exp "federated states of micronesia"/ or fiji/ or gabon/ or gambia/ or exp "georgia (republic)"/ or ghana/ or grenada/ or guatemala/ or guinea/ or guinea-bissau/ or guyana/ or haiti/ or honduras/ or exp india/ or exp indonesia/ or iran/ or exp iraq/ or jamaica/ or jordan/ or kazakhstan/ or kenya/ or kiribati/ or kosovo/ or kuwait/ or kyrgyzstan/ or laos/ or lebanon/ or liechtenstein/ or lesotho/ or liberia/ or libyan arab jamahiriya/ or madagascar/ or malawi/ or exp malaysia/ or maldives/ or mali/ or malta/ or mauritania/ or mauritius/ or melanesia/ or moldova/ or monaco/ or mongolia/ or "montenegro (republic)"/ or morocco/ or mozambique/ or myanmar/ or namibia/ or nauru/ or nepal/ or nicaragua/ or niger/ or nigeria/ or niue/ or north africa/ or oman/ or exp pakistan/ or palau/ or palestine/ or panama/ or papua new guinea/ or paraguay/ or peru/ or philippines/ or polynesia/ or qatar/ or "republic of north macedonia"/ or romania/ or exp russian federation/ or rwanda/ or sahel/ or "saint kitts and nevis"/ or "saint lucia"/ or "saint vincent and the grenadines"/ or saudi arabia/ or senegal/ or exp serbia/ or seychelles/ or sierra leone/ or singapore/ or "sao tome and principe"/ or solomon islands/ or exp somalia/ or south africa/ or south asia/ or south sudan/ or exp southeast asia/ or sri lanka/ or sudan/ or suriname/ or syrian arab republic/ or taiwan/ or tajikistan/ or tanzania/ or thailand/ or timor-leste/ or togo/ or tonga/ or "trinidad andtobago"/ or tunisia/ or turkmenistan/ or tuvalu/ or uganda/ or exp ukraine/ or exp united arab emirates/ or uruguay/ or exp uzbekistan/ or vanuatu/ or venezuela/ or viet nam/ or western sahara/ or yemen/ or zambia/ or zimbabwe/

88 "organisation for economic co-operation and development"/

89 exp australia/ or "australia and new zealand"/ or austria/ or baltic states/ or exp belgium/ or exp canada/ or chile/ or colombia/ or costa rica/ or czech republic/ or denmark/ or estonia/ or europe/ or exp finland/ or exp france/ or exp germany/ or greece/ or hungary/ or iceland/ or ireland/ or israel/ or exp italy/ or japan/ or korea/ or latvia/ or lithuania/ or luxembourg/ or exp mexico/ or netherlands/ or new zealand/ or north america/ or exp norway/ or poland/ or exp portugal/ or scandinavia/ or sweden/ or slovakia/ or slovenia/ or south korea/ or exp spain/ or switzerland/ or exp united kingdom/ or "turkey (republic)"/ or exp united states/ or western europe/

90 european union/

91 developed country/

92 or/88-91

93 87 not 92

94 86 not 93 [non OECD countries removed]

95 exp juvenile/ not exp adult/

96 94 not 95 [Child studies removed]

97 (elder mistreat* or elder abuse* or elder neglect*).tw,kf.

98 elder abuse/

99 97 or 98

100 96 not 99 [Elder abuse studies removed]

**MEDLINE**

1 emergency medical services/ or advanced trauma life support care/ or call centers/ or emergency medical dispatch/ or emergency medical service communication systems/

2 exp airway management/ or exp emergency treatment/ or exp ambulatory care/ or exp critical care/ or exp perioperative care/ or exp preoperative care/

3 exp emergency service, hospital/ or emergency services, psychiatric/ or hotlines/ or poison control centers/ or exp "transportation of patients"/ or triage/ or critical care nursing/ or emergency nursing/

4 (emergenc* adj5 (care or service* or health* or ill* or treat* or medic* or unit* or centre* or centre* or department* or setting*)).tw,kf.

5 (acute* adj5 (care or service* or health* or ill* or treat* or medic* or unit* or centre* or centre* or department* or setting* or ward?)).tw,kf.

6 (trauma* adj5 (care or service* or ill* or unit* or centre* or centre* or department*)).tw,kf.

7 (ambula* adj5 (care or service* or unit* or centre* or centre* or department* or setting*)).tw,kf.

8 (critical* adj2 (care or ill*)).tw,kf.

9 (urgent adj3 (care or service* or medic*)).tw,kf.

10 "intensive care".tw,kf.

11 paramedic*.tw,kf.

12 or/1-11 [Acute Care or Ambulance services]

13 agonistic behavior/

14 exp bullying/

15 problem behavior/

16 exp harassment, non-sexual/

17 sexual harassment/

18 incivility/

19 Professional Misconduct/

20 Hostility/

21 bully*.tw,kf.

22 harass*.tw,kf.

23 intimidat*.tw,kf.

24 (lateral* adj2 violence).tw,kf.

25 (horizontal* adj2 violence).tw,kf.

26 (transgressive adj3 behavio?r*).tw,kf.

27 (disruptive adj3 behavio?r*).tw,kf.

28 (unprofessional adj3 behavio?r*).tw,kf.

29 (micro-aggress* or microaggress*).tw,kf.

30 incivil*.tw,kf.

31 uncivil*.tw,kf.

32 rude*.tw,kf.

33 mistreat*.tw,kf.

34 (professional* adj3 misconduct).tw,kf.

35 mobbing.tw,kf.

36 hazing.tw,kf.

37 (negative behavio* or negative act?).tw,kf.

38 (gaslight* or malic*).tw,kf.

39 (Hidden adj5 (aggressi* or abus* or violenc*)).tw,kf.

40 or/13-39 [Unprofessional behaviours]

41 exp Health Personnel/

42 exp Students, Health Occupations/

43 exp education, graduate/ or "internship and residency"/ or teaching rounds/

44 exp Interprofessional Relations/

45 exp Patient Care Team/

46 Workplace/

47 (nurs* or midwif* or midwiv*).tw,kf.

48 paramedic?.tw,kf.

49 (doctor? or physician? or clinician? or surgeon? or consultant?).tw,kf.

50 (student? adj2 (medic* or health* or clinic*)).tw,kf.

51 intern?.tw,kf.

52 resident?.tw,kf.

53 (Therapist? or Pharmacist? or Optometrist? or Nutritionist? or Dentist? or Physiotherapist?).tw,kf.

54 (Audiologist? or Anatomist? or Allergist? or An?esthetist? or An?esthesiologist? or Cardiologist? or Dieti#ian? or Endocrinologist? or Gastroenterologist? or GP? or Geriatrician? or Hospitalist? or Oncologist?).tw,kf.

55 (Ophthalmologist? or Otolaryngologist? or Pathologist? or P?ediatrician? or Physiatrist? or Psychiatrist? or Pulmonologist? or Radiographer? or Radiologist?).tw,kf.

56 medic?.tw,kf.

57 assistant?.tw,kf.

58 (cleaner? or ancillary or porter?).tw,kf.

59 (auxillary or auxillaries or administrator? or secretary or secretaries or receptionist? or technician?).tw,kf.

60 (employee? or worker? or Staff or personnel or practitioner? or professional? or workforce* or team*).tw,kf. /freq=2

61 (workplace* or "work place*" or worksite* or "work site*" or "work setting*").tw,kf.

62 or/41-61 [Staff]

63 40 and 62 [UB and Staff search 1]

64 physician-nurse relations/ or interprofessional relations/

65 aggression/ or prejudice/ or Social Discrimination/ or ageism/ or gender equity/ or homophobia/ or exp racism/ or sexism/ or weight prejudice/ or xenophobia/ or Perceived Discrimination/ or Coercion/

66 64 and 65 [Aggression or prejudice among staff MeSH]

67 ((staff or employee* or work* or nurs* or doctor?) adj8 (WPV or violen*) adj5 among*).tw,kf.

68 ((staff or employee* or work* or nurs* or doctor?) adj8 coerci* adj5 among*).tw,kf.

69 ((staff or employee* or work* or nurs* or doctor?) adj8 Victim* adj5 among*).tw,kf.

70 ((staff or employee* or work* or nurs* or doctor?) adj8 (humiliat* or hostil*)).tw,kf.

71 ((staff or employee* or work* or nurs* or doctor?) adj3 undermin*).tw,kf.

72 ((staff or employee* or work* or nurs* or doctor?) adj6 (discrimination or discriminatory or discriminated)).tw,kf.

73 "abusive supervision".tw,kf.

74 (workplace adj3 (conflict* or aggressi* or abus*)).tw,kf.

75 ((staff or employee* or work* or nurs* or doctor?) adj5 (gender adj2 (inequalit* or equalit*))).tw,kf.

76 ((staff or employee* or work* or nurs* or doctor?) adj5 (racism or racist or (racial adj3 abus*))).tw,kf.

77 ((staff or employee* or work* or nurs* or doctor?) adj5 (sexism or sexist)).tw,kf.

78 ((staff or employee* or work* or nurs* or doctor?) adj8 ((disabilit* or disabled) adj5 (inequalit* or equalit*))).tw,kf.

79 ((staff or employee* or work* or nurs* or doctor?) adj8 ableis*).tw,kf.

80 ((staff or employee* or nurs* or doctor?) adj1 (aggressi* or abus*)).tw,kf.

81 or/66-80 [UB and staff search 2]

82 63 or 81 [UB among Staff final search]

83 12 and 82 [UB among Staff in Acute Care]

84 afghanistan/ or africa/ or africa, northern/ or africa, central/ or africa, eastern/ or "africa south of the sahara"/ or africa, southern/ or africa, western/ or albania/ or algeria/ or andorra/ or angola/ or "antigua and barbuda"/ or argentina/ or armenia/ or azerbaijan/ or bahamas/ or bahrain/ or bangladesh/ or barbados/ or belize/ or benin/ or bhutan/ or bolivia/ or borneo/ or "bosnia and herzegovina"/ or botswana/ or brazil/ or brunei/ or bulgaria/ or burkina faso/ or burundi/ or cabo verde/ or cambodia/ or cameroon/ or central african republic/ or chad/ or exp china/ or comoros/ or congo/ or cote d'ivoire/ or croatia/ or cuba/ or "democratic republic of the congo"/ or cyprus/ or djibouti/ or dominica/ or dominican republic/ or ecuador/ or egypt/ or el salvador/ or equatorial guinea/ or eritrea/ or eswatini/ or ethiopia/ or fiji/ or gabon/ or gambia/ or "georgia (republic)"/ or ghana/ or grenada/ or guatemala/ or guinea/ or guinea-bissau/ or guyana/ or haiti/ or honduras/ or independent state of samoa/ or exp india/ or indian ocean islands/ or indochina/ or indonesia/ or iran/ or iraq/ or jamaica/ or jordan/ or kazakhstan/ or kenya/ or kosovo/ or kuwait/ or kyrgyzstan/ or laos/ or lebanon/ or liechtenstein/ or lesotho/ or liberia/ or libya/ or madagascar/ or malaysia/ or malawi/ or mali/ or malta/ or mauritania/ or mauritius/ or mekong valley/ or melanesia/ or micronesia/ or monaco/ or mongolia/ or montenegro/ or morocco/ or mozambique/ or myanmar/ or namibia/ or nepal/ or nicaragua/ or niger/ or nigeria/ or oman/ or pakistan/ or palau/ or exp panama/ or papua new guinea/ or paraguay/ or peru/ or philippines/ or qatar/ or "republic of belarus"/ or "republic of north macedonia"/ or romania/ or exp russia/ or rwanda/ or "saint kitts and nevis"/ or saint lucia/ or "saint vincent and the grenadines"/ or "sao tome and principe"/ or saudi arabia/ or serbia/ or sierra leone/ or senegal/ or seychelles/ or singapore/ or somalia/ or south africa/ or south sudan/ or sri lanka/ or sudan/ or suriname/ or syria/ or taiwan/ or tajikistan/ or tanzania/ or thailand/ or timor-leste/ or togo/ or tonga/ or "trinidad and tobago"/ or tunisia/ or turkmenistan/ or uganda/ or ukraine/ or united arab emirates/ or uruguay/ or uzbekistan/ or vanuatu/ or venezuela/ or vietnam/ or west indies/ or yemen/ or zambia/ or zimbabwe/

85 "Organisation for Economic Co-Operation and Development"/

86 australasia/ or exp australia/ or austria/ or baltic states/ or belgium/ or exp canada/ or chile/ or colombia/ or costa rica/ or czech republic/ or exp denmark/ or estonia/ or europe/ or finland/ or exp france/ or exp germany/ or greece/ or hungary/ or iceland/ or ireland/ or israel/ or exp italy/ or exp japan/ or korea/ or latvia/ or lithuania/ or luxembourg/ or mexico/ or netherlands/ or new zealand/ or north america/ or exp norway/ or poland/ or portugal/ or exp "republic of korea"/ or "scandinavian and nordic countries"/ or slovakia/ or slovenia/ or spain/ or sweden/ or switzerland/ or turkey/ or exp united kingdom/ or exp united states/

87 European Union/

88 Developed Countries/

89 or/85-88

90 84 not 89 [OECD search filter NICE 2021]

91 83 not 90 [UB among Acute Care Staff with non-OECD countries removed]

92 (exp Child/ or Adolescent/ or exp Infant/) not exp Adult/

93 91 not 92 [Child studies removed]

94 (elder mistreat* or elder abuse* or elder neglect*).tw,kf.

95 93 not 94 [Elder abuse studies removed]
